# Recyclable and eco-friendly xanthan gum-based hygroscopic hydrogel for atmospheric water harvesting

**DOI:** 10.1038/s41598-025-23971-3

**Published:** 2025-11-17

**Authors:** A’laa Mohamed Safwat, Mohamed Abd-Elzaher, I. H. Saleh, Moataz Soliman, Wagih Abdel‑Alim Sadik

**Affiliations:** 1 Department of Basic and Applied Sciences, Faculty of Engineering, Arab Academy for Science, Technology, and Maritime Transport, Alexandria, Egypt; 2 Institute of Graduate Studies & Research, Alexandria University, Alexandria, Egypt

**Keywords:** XG-g-PAA hydrogel, Microwave, Water harvesting, Grafting, Chemistry, Engineering, Environmental sciences, Materials science

## Abstract

Water scarcity is a pressing issue in several regions of the world. Nevertheless, atmospheric water vapor offers an abundant freshwater source that is constantly renewed through the hydrological cycle. Among water harvesting technologies, Sorption-based methods, which involve sorbents to capture water vapor and heat to release the sorbed water, have proven its effectiveness for collecting moisture. This study introduced a novel type of sorbent hydrogel made from natural, cost-effective, and non-toxic polysaccharide polymer. The hydrogel was prepared by grafting partially neutralized acrylic acid (AA) monomers onto Xanthan Gum (XG) using N, N′-methylene-bis-acrylamide (N-MBA) as a crosslinker and potassium persulfate as an initiator. Characterization was done using FTIR, SEM, TGA, and XRD. It was found that the swelled sample showed a higher value of water uptake which reached to about 50% of their maximum swelling capacity at the first 3 min. Moreover, the water uptake of the hydrogel increased as the amount of AA and CaCl_2_ increased up to a certain value and decreased beyond this value. While, increasing the concentration of N-MBA led to decreasing the water uptake. Finally, over 94% of the sorbed water being rapidly released. While, the hydrogel showed excellent reusability without noticeable fading.

**Supplementary Information:**

The online version contains supplementary material available at 10.1038/s41598-025-23971-3.

## Introduction

As we all know, water is the most plentiful resource on Earth and essential for all life forms as best described by Leonardo Da Vinci’s statement that “water is the driving force behind all of nature”^1^. Despite water covering 70% of the Earth’s surface, only 2.5% of this water is freshwater^2^. In light of rapid population growth, widespread clean water shortage, water resource contamination, and global climate change, the problem of freshwater shortage worsens each year and become one of the most pressing global challenges. Currently, a total of 470 million people is already facing severe water scarcity, and by 2050, it is predicted that more than two-thirds of the global population or six billion people on the planet will live in water scarce places^3,4^.

To address this challenge, researchers from all over the world have developed various effective water purification technologies to produce fresh water from nature and meet the ever-growing water demand, including seawater desalination, and atmospheric water harvesting (AWH), etc^2^.

Seawater desalination like reverse osmosis is considered to be one of the most common and effective way to produce fresh water. However, these processes face significant barriers such as high costs of investment, excessive energy consumption, the negative impact of disposing concentrated effluent on the ecosystem and marine life, limited permeability, and fouling. Moreover, these technologies usually require both centralized infrastructures and existence of water body, making it difficult to be applied in rural areas away from coastal line^5,6^.

On the other hand, the Earth’s atmosphere is full of water, which is regarded as a renewable water resource owing to the global hydrological cycle. Approximately 12,900 cubic kilometers of water, representing about 10% of the total freshwater in rivers and lakes, is estimated to be present in the atmosphere at any moment^7,8^.

The idea of harvesting water from the air is nothing new. In nature, numerous creatures can live and reproduce in extremely dry environments due to their unique abilities. For example, the Namib Desert beetle demonstrates exceptional water collection capabilities. It captures and combines water droplets on the hydrophilic areas and facilitates their runoff using the hydrophobic regions^9^. Therefore, these strategies and mechanisms, which inspired from nature, can be integrated into materials science for developing materials and equipment with high water harvesting efficiency.

Without a doubt, atmospheric water can be harvested through numerous methods. But, the most known ways used to harvest water from the atmosphere are fog harvesting, dewing, and sorption-based AWH^10^.

Fog harvesting and dewing are the most ancient and widely used methods. However, these methods are impractical and inaccessible for everyday use due to their limitations^11^.

Conversely, sorption-based AWH depends on using of dehydrated materials (sorbents) with high affinity for moisture to harvest atmospheric water vapor and concentrate them on the surface or internal volume at night. During daylight hours, the captured water can be released through heating, condensed, and collected as liquid water^12,13^.

The sorption method’s primary advantage is its independence from regional factors and its effective performance over a wide range of humidity levels, making it adaptable for water production in different environments using a variety of sorbents^12^.

Several sorbents with large pore volume and high surface area have been employed for extracting water from the air, including zeolite, silica gel, metal–organic frameworks (MOFs), hygroscopic salts, and composite sorbents^14^.

Zeolite and silica gels which are known for their low cost, non-toxicity, and large surface areas exhibit vapor adsorption capabilities across a broad range of humidity levels. However, it becomes difficult to regenerate water molecules after adsorption due to the strong bonding with water molecules (>160 °C), which limit their opportunity to be employed as atmospheric water harvesters^15^.

MOFs include high adsorption capacity, porosity, biocompatibility, and availability of various metal centers and ligands which provide MOFs with diverse structures and functions. Nonetheless, the high raw material costs, the complexity of their fabrication process, and concerns regarding the toxicity of organic ligands and metals hinder their widespread manufacturing and use^14,16^.

On the contrary, hygroscopic salts like LiCl and CaCl_2_, offer significant advantages such as high-water absorption capacity across a wide range of relative humidity levels, low price, availability, and environmental friendliness, making them attractive sorbents for AWH in arid areas. However, exposure to water vapor can cause hygroscopic salts to dissolve, leading to problems with particle agglomeration and the formation of passivation layers on the particle surfaces, which reduces the permeability of water vapor, sorption capacity, and difficulty in recovering the sorbents^17^.

To address this challenge, the most preferred approach is to integrate the hygroscopic salt into a designed matrix such as hydrogel, in which the loaded salt is responsible for harvesting water vapors while the hydrogel prevents the salts from dissolving in the absorbed water^18^.

Hydrogels or superabsorbent polymers are three-dimensional hydrophilic homopolymer or copolymer networks synthesized from natural and/or synthetic monomer or polymer materials. Due to their physically and/or chemically crosslinked structures and presence of functional groups such as hydroxyl (-OH), carboxyl (-COOH), sulfonic (-SO_3_H), and amide (-CONH_2_) groups, these materials have the ability to swell and retain 20–95% of water without dissolution^19–22^.

Microwave-assisted grafting of polysaccharides involves using microwave irradiation to facilitate the attachment of functional groups or polymers onto the polysaccharide backbone. This method offers advantages such as shorter reaction times, higher yields, and improved control over the grafting process compared to conventional methods^23^.

Our research aims to synthesize a cost-effective, fast synthesis (few seconds) and efficient Xanthan gum-g-poly acrylic acid (XG-g-PAA) hydrogel from ecofriendly, biodegradable polysaccharide (xanthan gum) using microwave-assisted grafting and evaluate its effectiveness in harvesting atmospheric water vapor from the atmosphere.

In this study, different degrees of grafting of xanthan gum with acrylic acid were successfully synthesized to obtain a hydrogel with high efficiency for harvesting the atmospheric water across a range of relative humidity of (60–80%). In addition, XG-derived hygroscopic hydrogel was also fabricated through incorporation of CaCl_2_ into the hydrogels by means of freeze drying to increase the amount of harvested water.

## Experimental work

### Materials

Xanthan gum (XG) which is a high molecular weight extracellular heteropolysaccharide with cellulose like backbone and produced by the microorganism *Xanthomonas campestris* was purchased from Loba Chemie, Mumbai – India. Acrylic acid (AA) with an assay of 98%, was purchased from ADVENT company, India. N, N’- methylene-bis-acrylamide (N-MBA) crosslinker with an assay of 99% was purchased from CDH, New Delhi–India. Potassium persulfate (KPS) initiator was purchased from PIOCHEM, Egypt with an assay of 99%. Anhydrous calcium chloride (CaCl_2_) and methyl alcohol with an assay of 99.5% were purchased from Chemajet, Egypt. Sodium hydroxide (NaOH) was purchased from El-Gomhoria, Egypt. All the chemicals were used as received without further purification. For all the experiments, distilled water was used.

### Methods

The preparation processes of grafted hydrogel, as well as calcium chloride incorporated grafted hydrogel were illustrated in details according to the following procedures.

#### Preparation of xanthan gum-g-poly acrylic acid hydrogel

XG-g-PAA hydrogel was synthesized by dissolving a certain amount of sodium hydroxide in 15 ml distilled water using a magnetic stirrer in a 150 ml beaker. Then, different amounts of AA (5, 7.5, and 10 ml) were added dropwise with cooling. Afterward, 0.3 g of potassium persulfate (KPS) was added with continuous stirring followed by addition of precalculated amounts of N-MBA (0.05, 0.1, and 0.2% of the total weight) while maintaining constant and vigorous stirring. After complete dissolution, 1 gram of XG powder was added gently with keeping vigorous stirring until a homogenous solution was obtained. The reaction vessel was subsequently placed in a domestic microwave oven and irradiated at a power of 800 W with periodical pausing and cooling at (~ 65 °C) of the reaction mixture. The temperature of the reaction was controlled by the thermometer to eliminate the formation of any homopolymer. This microwave irradiation-cooling cycle was continued for up to 2 min until a thick gel-like substance was formed (see Supplementary Fig. S1). The reaction vessel and its contents were cooled to stop the grafting reaction. After allowing the prepared hydrogels to cool to room temperature, they were washed several times with methanol: water mixture (7:3; V: V) to remove unreacted monomers and adhered homopolymer. Finally, the hydrogel was filtered, cut to small pieces, dried at 60 °C till constant weight, and then grinded. The prepared hydrogel is named as XG-g-PAA hydrogel^24,25^.

#### The grafting percentage (%G) and grafting efficiency (%GE)

The grafting of AA on XG was evaluated by calculating the percentage of grafting (% G) and the percentage of grafting efficiency (% GE) for the synthesized hydrogel using the following equation^26^:

1 $${\text{\% Grafting~}}\left( {{\text{\% G}}} \right)=\left( {\frac{{{{\text{w}}_3} - {{\text{w}}_0}}}{{{{\text{w}}_0}}}} \right) \times 100$$

2 $${\text{\% ~Grafting~efficiency~}}\left( {{\text{\% GE}}} \right)=\left( {\frac{{{{\text{w}}_3} - {{\text{w}}_0}}}{{{{\text{w}}_1}+{{\text{w}}_2}}}} \right) \times 100$$

Where: W_0_, W_1_, W_2_, and W_3_ are the masses of XG or GG, sodium acrylate, unneutralized AA, and XG-g-PAA or GG-g-PAA hydrogel respectively.

#### Preparation of calcium chloride incorporated XG-g-PAA hydrogel

After following the same procedures mentioned above, the as-prepared hydrogel (i.e. XG-g-PAA hydrogel) was filtered and placed 12 h in the refrigerator for pre-freezing. Then, it was freeze-dried at − 80 °C for 48 h. After that, the freeze-dried hydrogel was immersed into 10 ml of CaCl_2_ solution with varied concentrations (i.e., 0.4, 0.6, and 0.8 g/ml) for 48 h under ambient conditions. The resulted hydrogel was gently dried with filter paper and dried at 80 °C in an oven for 3 days followed by grinding and finally named as CaCl_2_ incorporated XG-g-PAA hydrogel and stored in a desiccator for usage^27,28^.

The preparation procedures for the grafted hydrogel as well as CaCl_2_ incorporated hydrogel were illustrated in Supplementary Fig. S2. Also, the different grades of the grafted XG hydrogel were summarized in Table 1.

**Table 1:** The different grades of the synthesized XG-g-PAA hydrogel.

Grade	Xanthan gum (XG)	Acrylic acid (AA)	Sodium hydroxide (NaOH)	*N*, *N*′- methylene-bis-acrylamide (MBA)		Potassium persulfate (KPS)
	Weight (g)	Volume (ml)	Weight (g)	Weight (g)	%	Weight (g)
XG-g-PAA 1	1	5	2.08	0.0042	0.05	0.3
XG-g-PAA 2	1	7.5	3.12	0.0060	0.05	0.3
XG-g-PAA 3	1	10	4.16	0.0077	0.05	0.3
XG-g-PAA 1’	1	5	2.08	0.0084	0.1	0.3
XG-g-PAA 2’	1	7.5	3.12	0.01192	0.1	0.3
XG-g-PAA 3’	1	10	4.16	0.0155	0.1	0.3
XG-g-PAA 1’’	1	5	2.08	0.0168	0.2	0.3
XG-g-PAA 2’’	1	7.5	3.12	0.0238	0.2	0.3
XG-g-PAA 3’’	1	10	4.16	0.0309	0.2	0.3

#### Characterization of the prepared hydrogels

The synthesized hydrogels, XG-g-PAA and CaCl_2_ incorporated XG-g-PAA hydrogels, were characterized in order to investigate their morphology, structure, chemical composition, and thermal stability.

#### Fourier transforms infrared spectroscopy (FTIR)

The chemical functional groups of XG, AA, and XG-g-PAA hydrogel were identified by FTIR. The spectra were obtained over a wavenumber range of 4000 –500 cm^− 1^. With respect to XG and XG-g-PAA hydrogel, characterization was carried out in solid state using KBr pellets. The samples were pulverized in a mortar and homogenized with KBr followed by pressing the mixture in a hydraulic press to cast pellets to thin discs. While for AA, the sample was prepared by using few drops between two discs of NaCl. All samples are characterized using spectrophotometer (Perkin Elmer Spectrum BX, USA) at Institute of Graduate Studies and Research, Alexandria University.

#### Scanning electron microscopy (SEM)

The structural morphologies of the prepared hydrogel were employed using SEM of JEOL model JSM-6010 LV (Japan) at Faculty of Science, Alexandria University. With SEM, several images were taken for different areas to inspect the porosity of the prepared hydrogel. Prior to SEM analysis, all samples were mounted on the specimen stabs and coated with thin film of gold by the ion sputtering electrode to induce the conduction effect since the samples were non-conductive, then introduced into equipment on their holder. After that equipment parameters like accelerated voltage, focusing, working distance, etc. were adjusted for every sample to get the best images.

#### Thermogravimetric analysis (TGA)

TGA was performed to measure the thermal stability of the prepared hydrogel by monitoring their weight loss as a function of temperature using thermal analyzer (SDTQ600, TA instruments, USA) at Institute of Graduate Studies and Research, Alexandria University equipped with thermal analysis software in a nitrogen atmosphere. Samples of approximately 6 mg were heated in an aluminum cell from ambient temperature to 700 °C with a heating rate of 10 °C/min.

#### X-ray diffraction (XRD)

The incorporation of CaCl_2_ into the prepared hydrogel was investigated by XRD using Bruker D2 phaser diffractometer (Cu Kα, the wavelength is 1.5435 Å). The scanning completed for 2θ angles from 1 to 100, with a step time of 2 s and a step size of 0.02.

### Swelling behavior of XG-g-PAA and GG-g-PAA hydrogels

The swelling behavior of XG-g-PAA hydrogel was examined to confirm the formation of the prepared hydrogel. It was anticipated that properly formed hydrogel would swell instead of dissolving, while improper formation might result in the formation of a viscous solution known as an organogel^29^. Therefore, the water sorption of the hydrogel was investigated by conventional procedures. A known weight of the dried hydrogel (0.1 g) was placed in 250 ml beaker containing 100 ml of distilled water at room temperature. At pre-determined time intervals (5, 10, 15,………, 60 min), the swollen hydrogel was filtered by a stainless-steel strainer of 30 meshes and the water droplets adhered to surface were removed by blotting paper. After that, the increase in the weight of the samples was recorded and the measurements were made until the weight of swollen hydrogel reached a constant value. The swelling ratio at time (t) was calculated using the following equation:

3 $${\text{Swelling~ratio~}}\left( {{\text{g}}/{\text{g}}} \right)=\frac{{{{\text{W}}_{\text{t}}} - {{\text{W}}_{\text{d}}}}}{{{{\text{W}}_{\text{d}}}}}$$

Where W_t_ and W_d_ are the masses in grams of swollen hydrogel after a given time and the dry hydrogel respectively^28^.

### Water sorption setup description

**Fig. 1:**
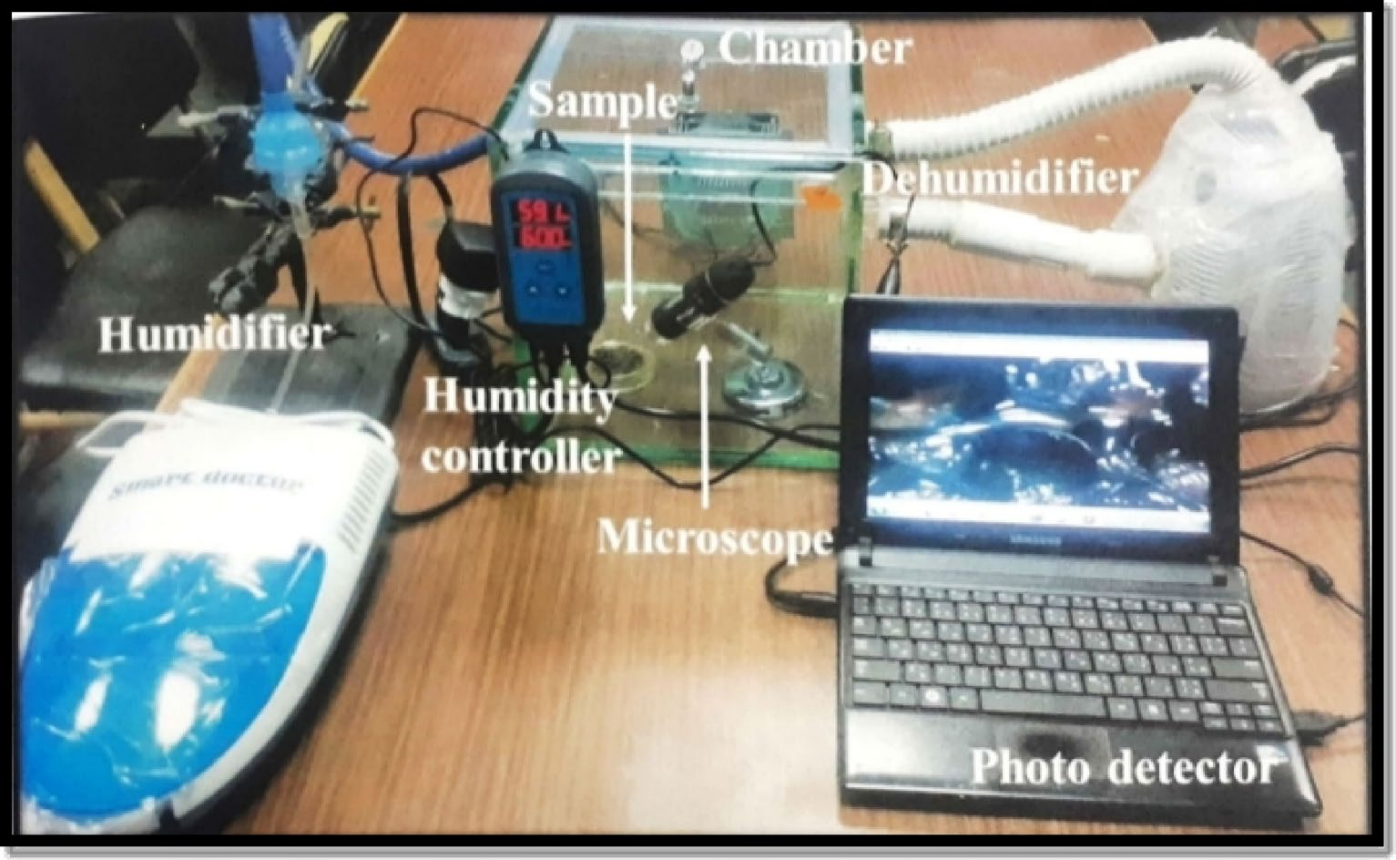
Digital photograph of the experimental setup used for AWH.

To perform the water vapor sorption test, a glass chamber of thickness 10 mm was fabricated with dimensions (25 * 25 * 25 cm) as shown in Fig. 1. Then, the synthesized hydrogel were placed on an aluminum foil at the bottom of the chamber where they will begin to sorb the water vapor present in the chamber. In order to simulate the water vapor inside the chamber, a humidifier has been used to generate water vapor. Also, to adjust the humidity value inside the chamber at a specific value (60, 70, and 80%), a humidity sensor (Inkbird Humidity Controller IHC200) was mounted in the test chamber and monitored the humidity in the chamber during the test. When the humidity deviated from the set value, the humidity detector would actuate the pump to humidify or dehumidify the air in the system. The switch modes between humidification (humidifier) and dehumidification (dehumidifier) were operated automatically to keep constant relative humidity value inside the chamber. Optical analysis of water vapor harvesting using Cooling Tech digital microscope (magnification range 1X ~ 1600X) was applied to record the process of collecting water by the sorbent after the harvesting process.

### Atmospheric water sorption experiment

The ability of synthesized hydrogels to capture water vapors was examined in an artificial climate chamber at room temperature and various levels of relative humidity (60–80%).

Before each sorption test, the tested sample was heated in an oven at 80 °C for at least 2 h to remove all the water that might be present in the sample. Then the weight of the dry sample was recorded before placing it into a constant temperature and humidity chamber. To fully expose the dried hydrogel to the moist air, it was placed on an aluminum foil and set in humid air with varying the relative humidity (RH) until full saturation was achieved (8 h). During the sorption process, the mass change of the synthesized hydrogels was measured at regular and pre-determined time intervals (2 h) during the water vapors sorption experiments using a high precision electronic balance (Quintix124, Sartorius, Germany) with an accuracy of 0.0001 g.

The water uptake capacity of the prepared hydrogels after moisture sorption was calculated using Eq. (3)^30,31^.

During the water vapor uptake experiment, the experimental data were repeated three times and the average value was calculated.

### Factors affecting water sorption by XG-g-PAA hydrogel

#### Crosslinker content (N-MBA)

Crosslinking density is considered to be an important swelling control element that must be taken into account through the preparation process of the hydrogels where some small changes can have a significant impact on altering the properties of the synthesized hydrogels. Therefore, to study the effect of crosslinker content on the swelling ratio of the prepared hydrogel as well as the amount of harvested water, series of three samples were prepared. In these samples the content of N-MBA crosslinker was varied from 0.05 to 0.2% of the total weight at constant amount of gum (XG), monomer (AA), and initiator (KPS) over 8 h periods.

#### Monomer content (AA)

Grafting has a crucial role in enhancing the sorption efficiency of the grafted sorbent. So, the main target of grafting of AA monomers on XG chains is to enhance the sorption efficiency of the synthesized XG-g-PAA hydrogel in order to increase the amount of harvested water. Therefore, the influence of grafting of AA on the hydrogel’s sorption efficiency as well as the amount of harvested water was investigated by synthesizing another series of three samples with varying the amount of AA monomer between (5.0–10 ml) while keeping the amount of the other parameters like gum (XG), initiator (KPS), and crosslinker (N-MBA) constant over 8 h periods.

#### Hygroscopic salt content (CaCl_2_)

In order to examine the sorption behavior of the prepared XG-g-PAA hydrogel after addition of CaCl_2_ and determine the optimum amount of CaCl_2_, further three samples were synthesized with different concentrations of CaCl_2_ (0.4, 0.6, and 0.8 g/ml) keeping the other influencing conditions such as the amount of gum (XG), initiator (KPS), monomer (AA), and crosslinker (N-MBA) constant.

### Atmospheric water desorption experiment

The optimum sample were placed in a constant temperature and humidity chamber at room temperature and 80% RH for 8 h to saturate it with moisture. After that, when the sorption test is finished, saturated sample was placed immediately into a glass dish and exposed to the heat in the oven which adjusted to different temperatures (60, 80, and 100 °C). After adjusting the heat, the mass of the hydrated sample was recorded gradually by an electronic balance at different desorption time intervals of 15, 30, 45, ………etc min. The percentage of evaporation efficiency was calculated using the following equation:

4 $${\text{Evaporation~Efficiency~}}\left( {{\eta _{\text{e}}}{\text{\% }}} \right)=\frac{{{{\text{m}}_{\text{b}}} - {{\text{m}}_{\text{c}}}}}{{{{\text{m}}_{\text{d}}}}} \times 100$$

Where, m_b_ is the mass in grams of the hydrogel after 8 h moisture sorption, m_c_ is the mass in grams of the hydrogel after various time intervals of vapor evaporation when subjected to heat, and m_d_ is the mass in grams of water sorbed by the hydrogel after 8 h moisture sorption^27,31^.

### Reusability of the prepared hydrogels

The hydrothermal stability of the prepared hydrogels was investigated as mentioned by Shi et al.^32^ The completely dry samples were placed in the artificial humidity and temperature chamber with a relative humidity of 80% for 8 h. It was then heated in an oven at 100 °C for 90 min. The obtained sample can be treated as a reference value to estimate the stability of atmospheric water hydrogel. The whole cycle was repeated for 12 times.

## Results and discussion

### Synthesis mechanism of XG-g-PAA hydrogel

**Fig. 2:**
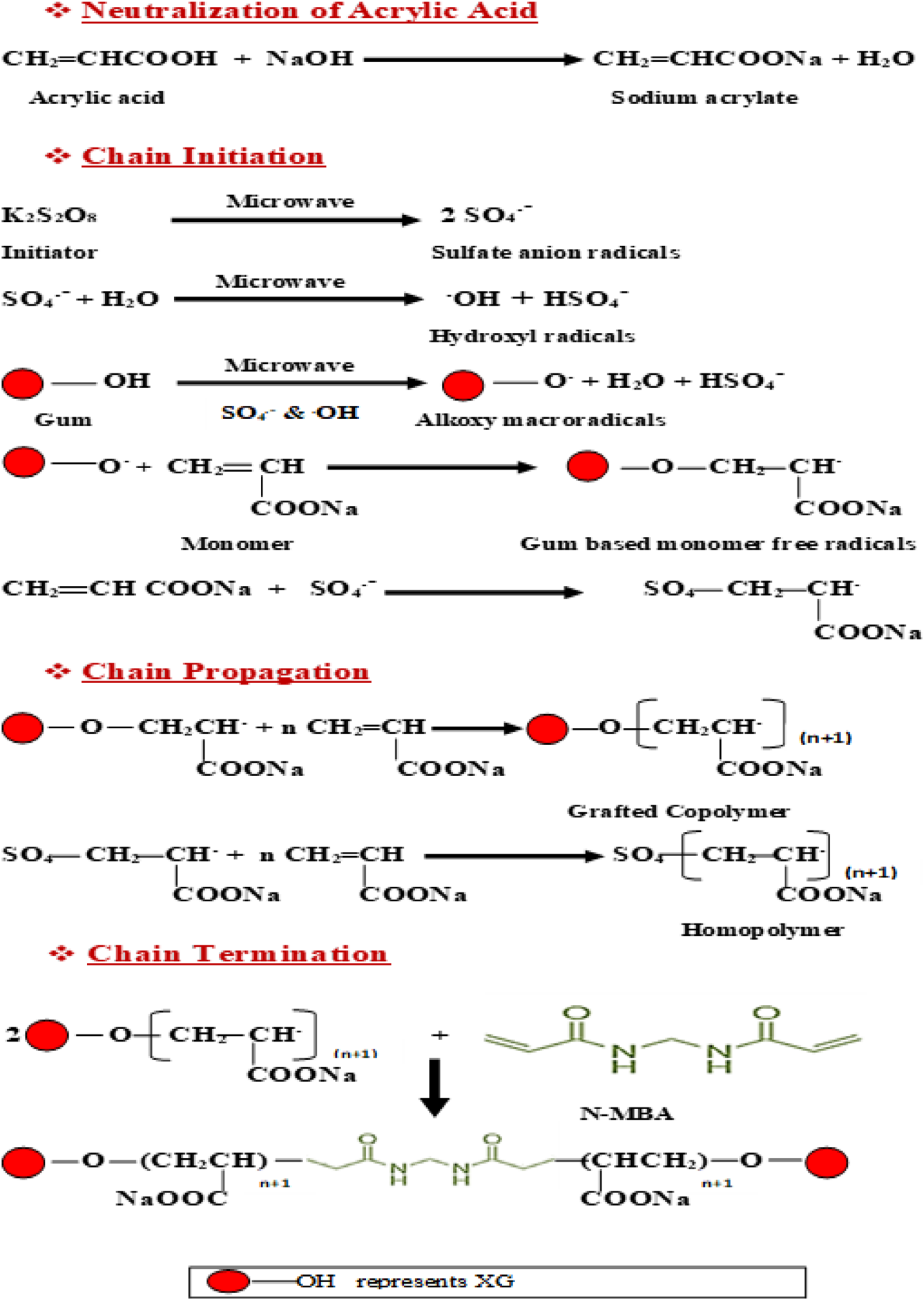
The proposed mechanism for XG-g-PAA hydrogel synthesis.

The proposed mechanism for the grafting process of AA onto XG backbone through microwave assisted free radical graft copolymerization method using KPS as free radical initiator and N-MBA as a crosslinker is illustrated in Fig. 2.

it can be concluded from Fig. 2 that, the hydroxyl groups of XG are the key and active sites for graft copolymerization of synthetic monomers.

**Fig. 3:**
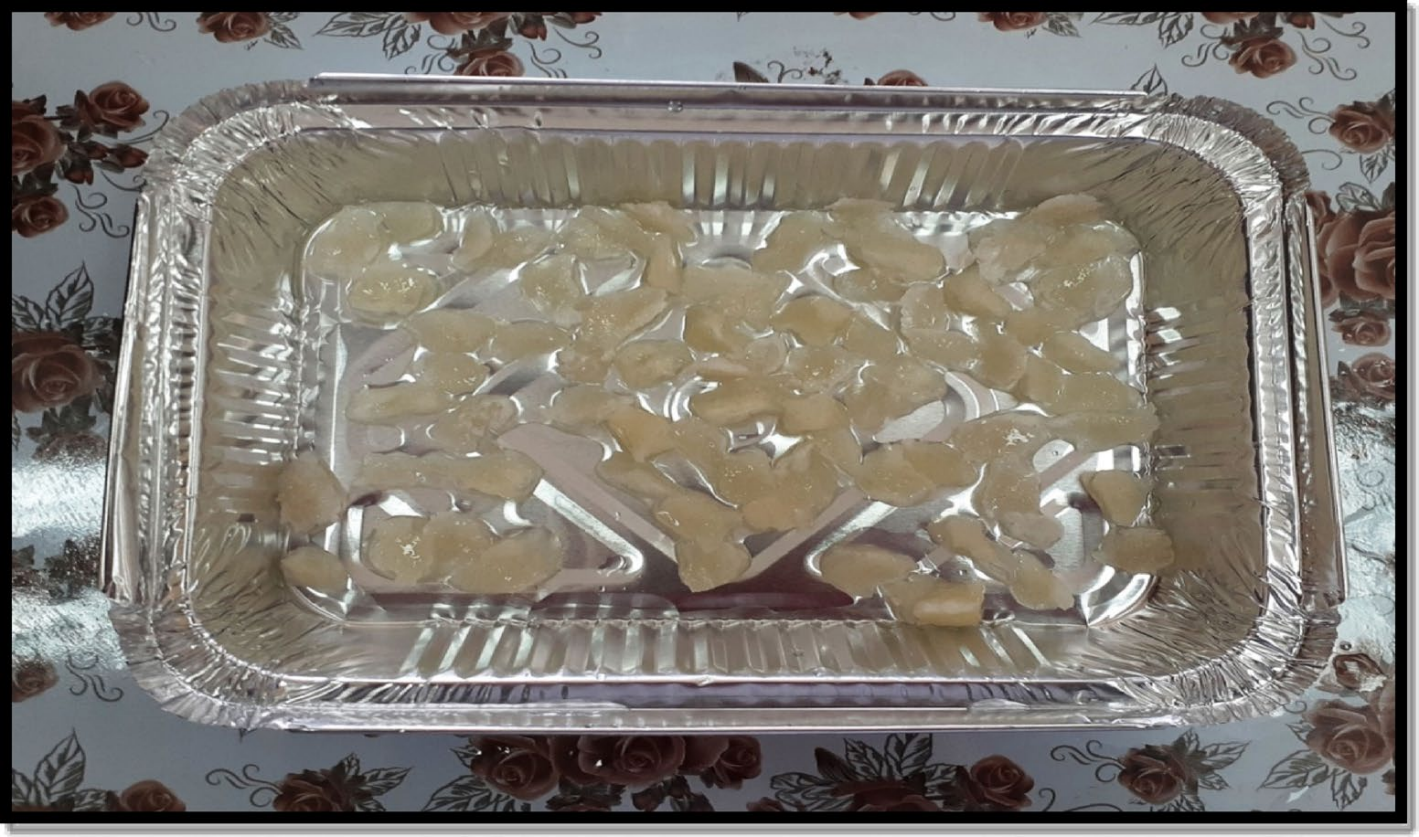
The photographs of the prepared XG-g-PAA hydrogel.

Figure 3 shows that the prepared hydrogel (i.e. XG-g-PAA) is translucent, soft, and elastic with a slippery surface.

### Evaluation of % G and % GE for XG-g-PAA hydrogel

The amount of AA grafted on XG backbone with respect to the mass of XG was estimated by calculating the percentage of grafting (%G). In addition, the amount of actually grafted AA to XG with respect to initial mass of AA was evaluated by calculating the percentage of grafting efficiency (% GE).

The various grades of XG-g-PAA hydrogel are evaluated in terms of (% G) and (% GE) and the obtained results are listed in Tables 2, 3 and 4.

From Tables 2, 3 and 4, it can be observed that as the volume of AA increased from 5 to 10 ml the (%G) increased from 582.38 to 1225.71 at 0.05% N-MBA, from 521.77 to 1166.75 at 0.1% N-MBA, and from 567.68 to 1202.19 at 0.2% N-MBA respectively. Also, the (% GE) increased from 94.9 to 99.7 at 0.05% N-MBA, from 85.9 to 94.9 at 0.1% N-MBA, and from 92.5 to 97.8 at 0.2% N-MBA respectively.

The increase in % G and % GE is attributed to the usage of microwave irradiation which enhances the generation of macroradicals. Therefore, increasing the possibility of molecular collision and hence grafting. As a result, the propagation reaction was enhanced and led to the formation of hydrogel. So, it is clear that the rapid microwave heating rate makes the grafting more productive and facile^33^.

**Table 2:** The (% G) and (% GE) of various grades of XG-g-PAA hydrogel at 0.05% N-MBA.

Grade	Amount					(% G)	(% GE)
	XG [W_0_ (g)]	A A [ml]	SA [W_1_ (g)]	AA (unneutralized) [W_2_ (g)]	XG-g-PAA hydrogel [W_3_ (g)]		
XG-g-PAA (1)	1	5	4.89	1.25	6.82	582.38	94.9
XG-g-PAA (2)	1	7.5	7.34	1.88	10.14	914.41	99.2
XG-g-PAA (3)	1	10	9.79	2.5	13.26	1225.71	99.7

**Table 3:** The (% G) and (% GE) of various grades of XG-g-PAA hydrogel at 0.1% N-MBA.

Grade	Amount					(% G)	(% GE)
	XG [W_0_ (g)]	A A [ml]	SA [W_1_ (g)]	AA (unneutralized) [W_2_ (g)]	XG-g-PAA hydrogel [W_3_ (g)]		
XG-g-PAA (1’)	1	5	4.89	1.25	6.22	521.77	85.9
XG-g-PAA (2’)	1	7.5	7.34	1.88	9.45	845.19	91.7
XG-g-PAA (3’)	1	10	9.79	2.5	12.67	1166.75	94.9

**Table 4:** The (% G) and (% GE) of various grades of XG-g-PAA hydrogel at 0.2% N-MBA.

Grade	Amount					(% G)	(% GE)
	XG [W_0_ (g)]	A A [ml]	SA [W_1_ (g)]	AA (unneutralized) [W_2_ (g)]	XG-g-PAA hydrogel [W_3_ (g)]		
XG-g-PAA (1’’)	1	5	4.89	1.25	6.68	567.68	92.5
XG-g-PAA (2’’)	1	7.5	7.34	1.88	9.82	881.88	95.7
XG-g-PAA (3’’)	1	10	9.79	2.5	13.02	1202.19	97.8

### Characterization of the prepared XG-g-PAA hydrogel

#### FTIR spectroscopy

**Fig. 4:**
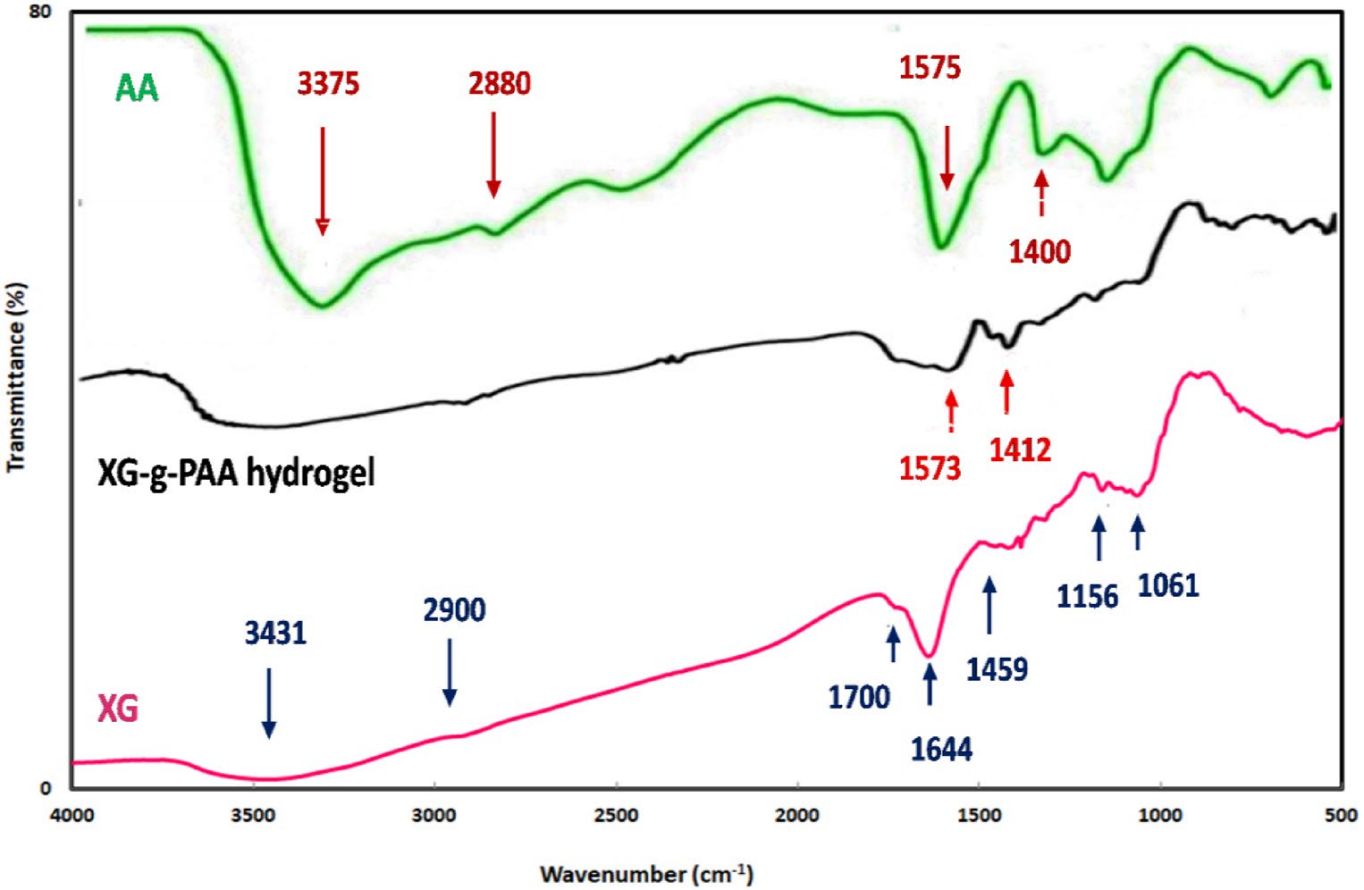
FTIR spectra of XG, AA, and XG-g-PAA hydrogel.

The characteristic peaks of XG and AA are elucidated in Table 5^34,37^. The absorption spectrum of the grafted hydrogel showed similar peaks to those of native XG with some differences and the existence of some additional peaks (Fig. 4). For instance, a new absorption peak was observed at 1573 cm^− 1^ which is related to (C = O) asymmetric stretching of AA. In addition, another new absorption peaks were recognized at 1412 which are related to (C = O) symmetric stretching that belongs to carboxylate anion of AA. These obtained results are consistent with the previously reported data^38,39^. Based on these observations, it can be concluded that the FTIR studies confirmed successful grafting of AA onto the XG backbone.

**Table 5:** The absorption peaks of native XG and AA.

Sample name	Wave number (cm^− 1^)	Peak	References
XG	3431	The primary and secondary hydroxyl groups (O–H)	^34,35^
	2900	Stretching vibration of the aliphatic alkyl group (-CH)	
	1700	Stretching vibration of the carbonyl (-C = O) in the acetyl group	
	1644	Asymmetric stretching vibration of carboxylate groups	
	1459	Symmetric stretching vibration of carboxylate (-COO^−^) group of glucuronic acid	
	1156	C-O bending	
	1061	Stretching vibration of glycosidic ether (C-O-C)	
AA	3375	Stretching vibrations of hydroxyl group	^36,37^
	2880	C–H stretching vibration	
	1575	C = O stretching vibration	
	1400	Symmetric stretching of CH_2_	

#### SEM

Based on the fact that scanning electron microscope offers a magnified view of the synthesized material that facilitates visual inspection and analysis, various scanning electron micrographs under different magnifications were taken in order to examine the surface morphology of XG, and XG-g-PAA.

**Fig. 5:**
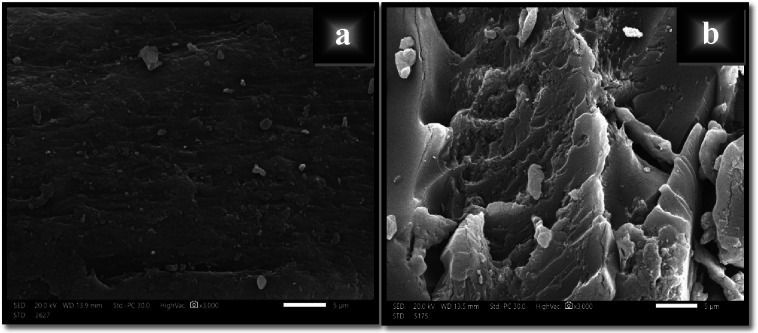
SEM morphology’s images of (a) native XG and (b) XG-g-PAA hydrogel.

**Fig. 6:**
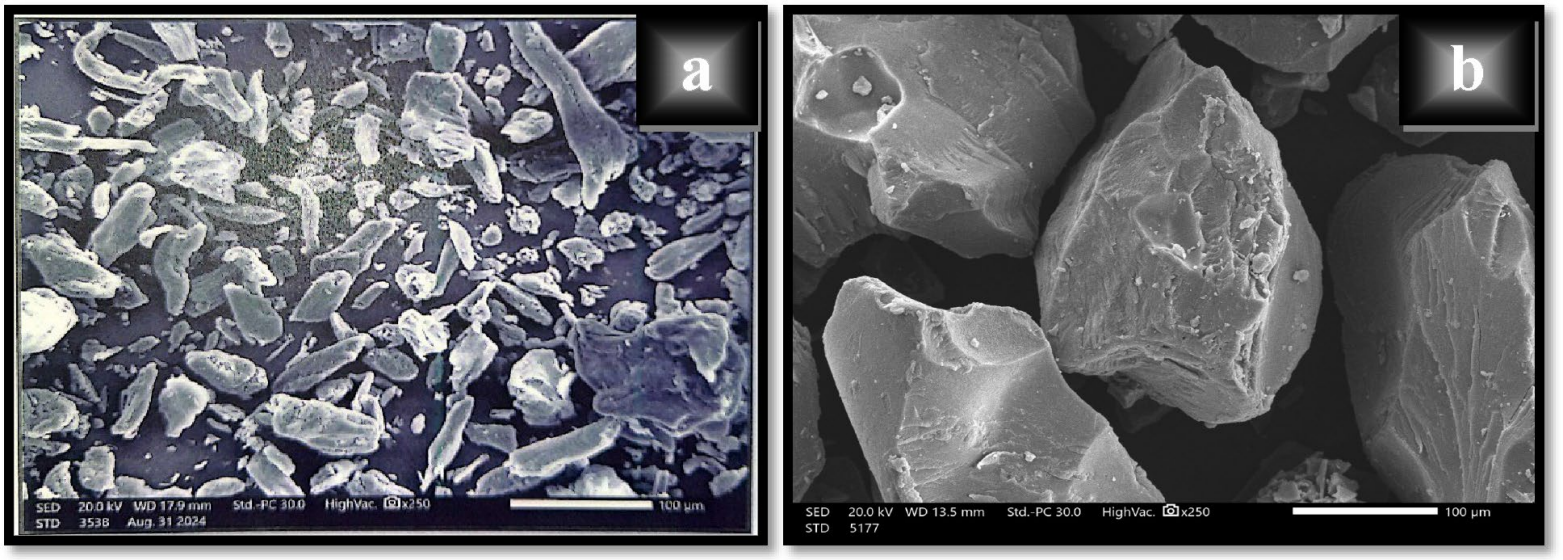
SEM particle size’s images of (a) native XG and (b) XG-g-PAA hydrogel.

The obtained SEM images indicated that the grafting of AA onto XG backbone resulted in a change in the morphology and size of XG particles as observed in Figs. 5 and 6. It is obvious that the smooth surface of native XG became rougher with more irregular shaped morphology and multiple folds after grafting as shown in Fig. 5. Moreover, the grafted sample showed a large number of pores and heterogeneous pore distribution which will enhance the sorption rate and allow the water’s diffusion around the sorption sites more rapidly. In addition, it is clear from Fig. 6 that XG-g-PAA particles have larger dimensions (in the range from 47.99 to 143.8 μm) compared to the native XG particles (in the range from 3.70 to 25.11 μm).

It is obvious that the irregular morphology with high heterogeneity displayed in case of XG-g-PAA hydrogel supports and confirms the grafting reaction. Also, the alteration in surface morphology was attributed to the formation of covalent bonds among various polymeric chains and crosslinking with N-MBA crosslinker. This observation is consistent with TGA analysis.

It is obvious that the irregular morphology with high heterogeneity displayed in case of XG-g-PAA hydrogel supports and confirms the grafting reaction. Also, the alteration in surface morphology was attributed to the formation of covalent bonds among various polymeric chains and crosslinking with N-MBA crosslinker. This observation is consistent with TGA analysis.

#### TGA

Based on the fact that the interaction between the polymer and the monomers affects the thermal stability of the synthesized hydrogel, it’s very important and useful to study and investigate the thermal properties of materials for selecting them for particular applications.

**Fig. 7:**
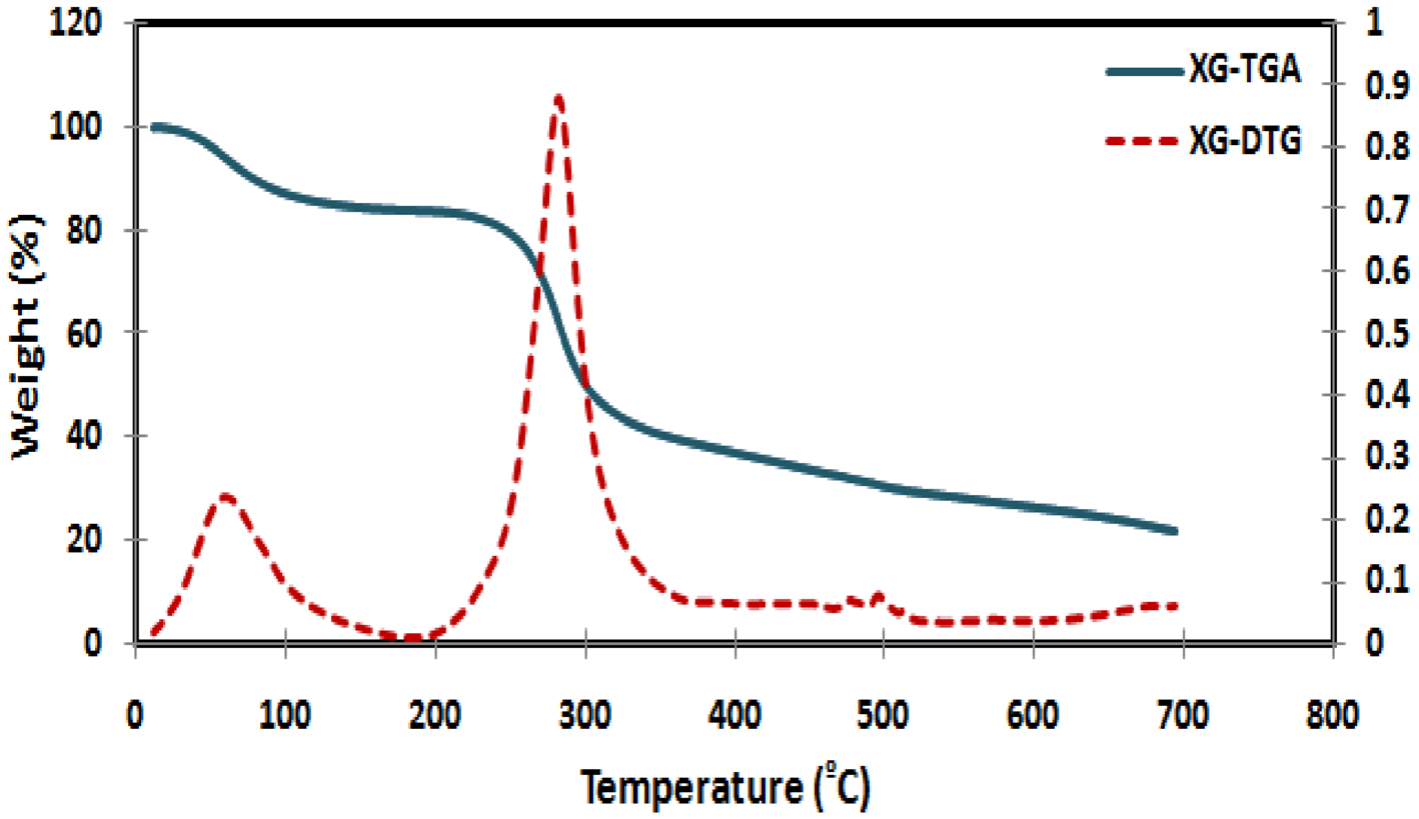
TGA and DTG of native XG.

As can be observed in Fig. 7, the TGA spectrum of native XG shows a two-step characteristic thermogram. The first stage weight loss took place within the temperature range from ambient temperature to 200 °C and was associated with the endothermic peak which could be attributed to the loss of moisture content present in the sample and represent about 16%. The second stage witnessed the major weight loss of about 63% which occurred during the temperature range from 200 to 400 °C and likely attributed to the breakdown of the polysaccharide backbone (XG). Finally, the rate of weight loss slows, and the residue at 700 °C was 21%.

**Fig. 8:**
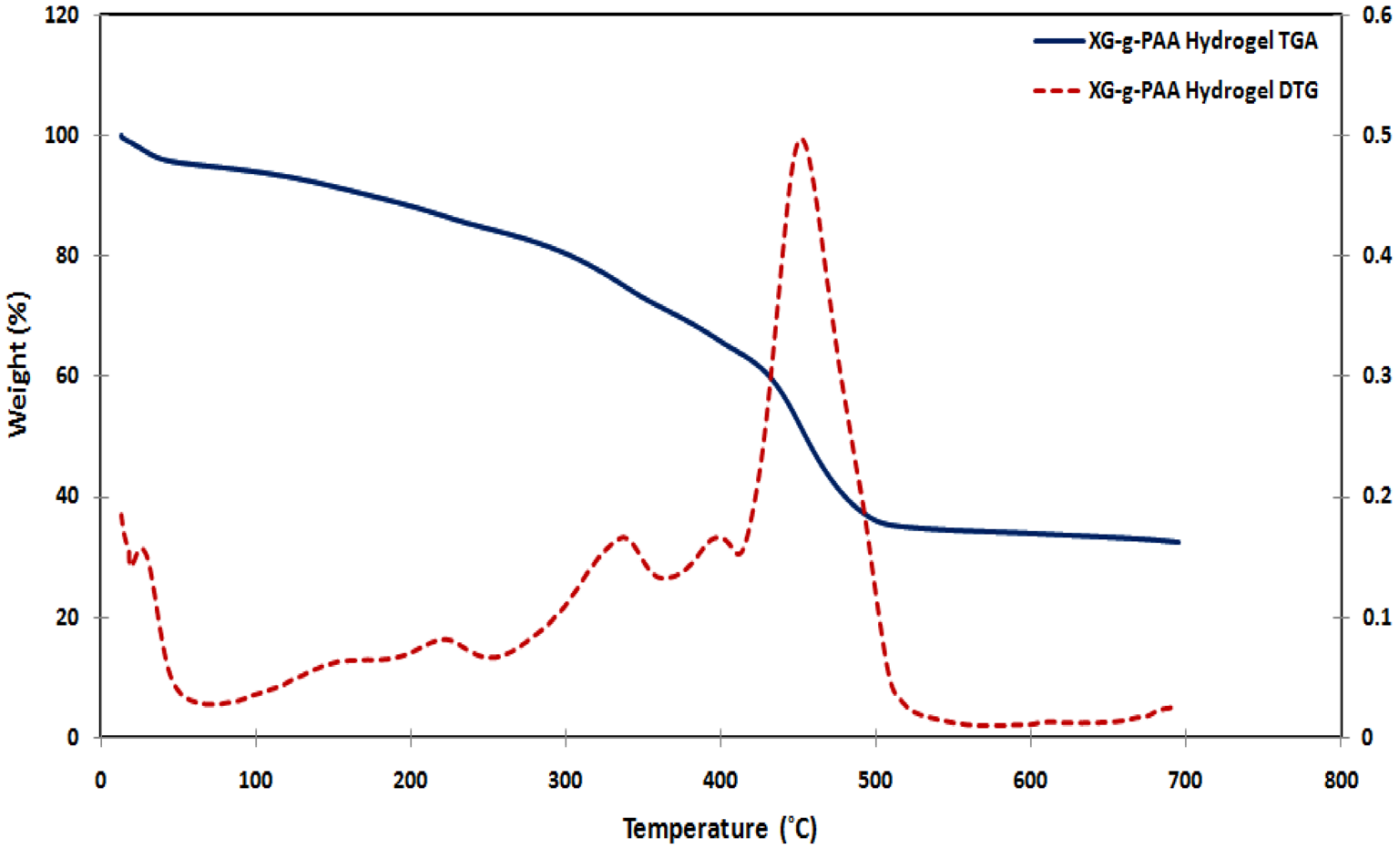
TGA and DTG of XG-g-PAA hydrogel.

With respect to grafted XG sample, it was observed that the thermogram of XG-g-PAA hydrogel exhibited a degradation pattern in 3 stages as shown in Fig. 8. The first weight loss (16%) occurred in the temperature range from ambient temperature to 251 °C and was attributed to elimination of adsorbed water to XG-g-PAA structure. The second stage of weight loss occurred over the range of 251–368 °C represented about 14% and may be related to the degradation of polysaccharide backbone as well as decomposition of carboxyl group of grafted PAA chains. Finally the third stage began at 368 and ended at 600 °C with a maximum weight loss of approximately 37% owing to degradation of AA network, which was grafted on the XG backbone and constructed crosslinking three-dimensional hydrogel network with XG. After 600 °C, the decomposition was extremely slow, with a weight loss of about 37%.

**Fig. 9:**
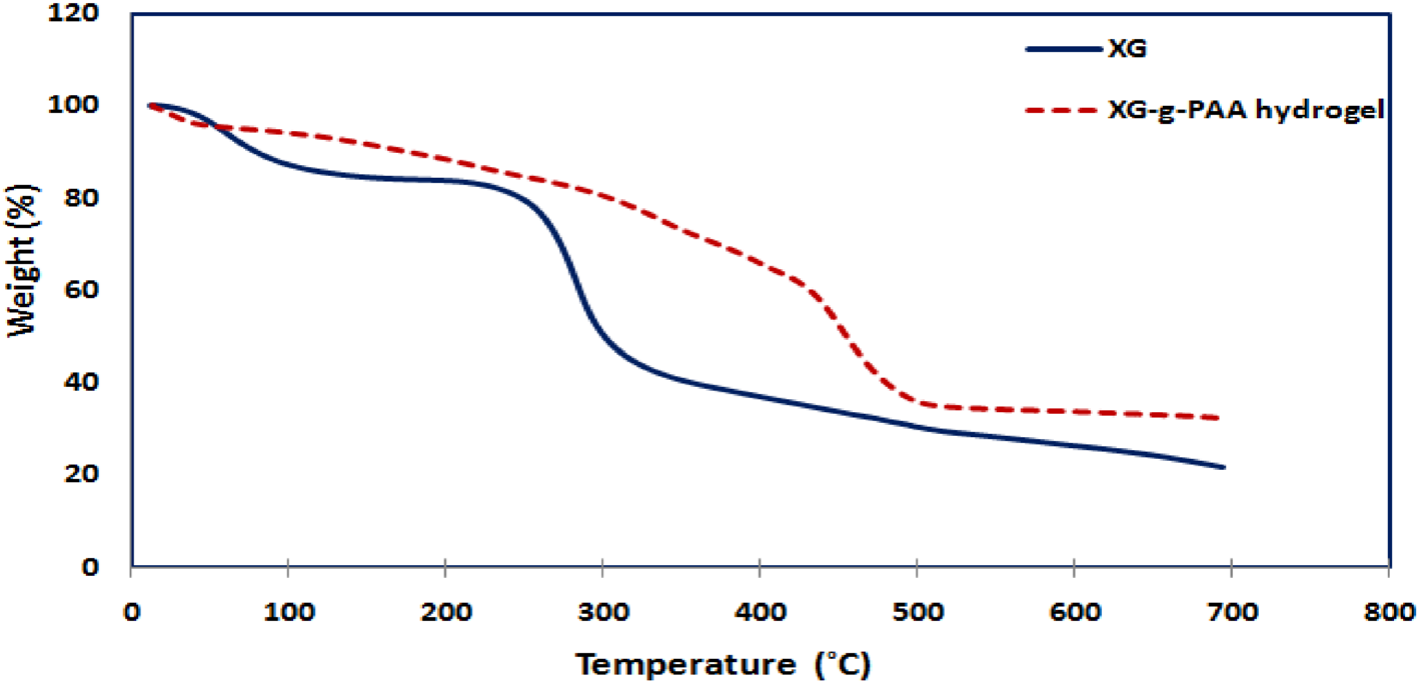
TGA of native XG and XG-g-PAA hydrogel.

From the obtained TGA curves of XG, and XG-g-PAA hydrogel Fig. 9., it was concluded that the grafting of AA and crosslinking have enhanced the thermal stabilities due to the creation of covalent bonds between polymeric chains within XG-g-PAA matrices. So, the synthesized hydrogels were found to be more thermally stable than native polysaccharide themselves. Moreover, the existences of an additional weight loss zone (3rd stage) in the grafted hydrogels are clear and strong evidence that PAA chains have been successfully grafted onto the backbone of XG.

#### XRD

**Fig. 10:**
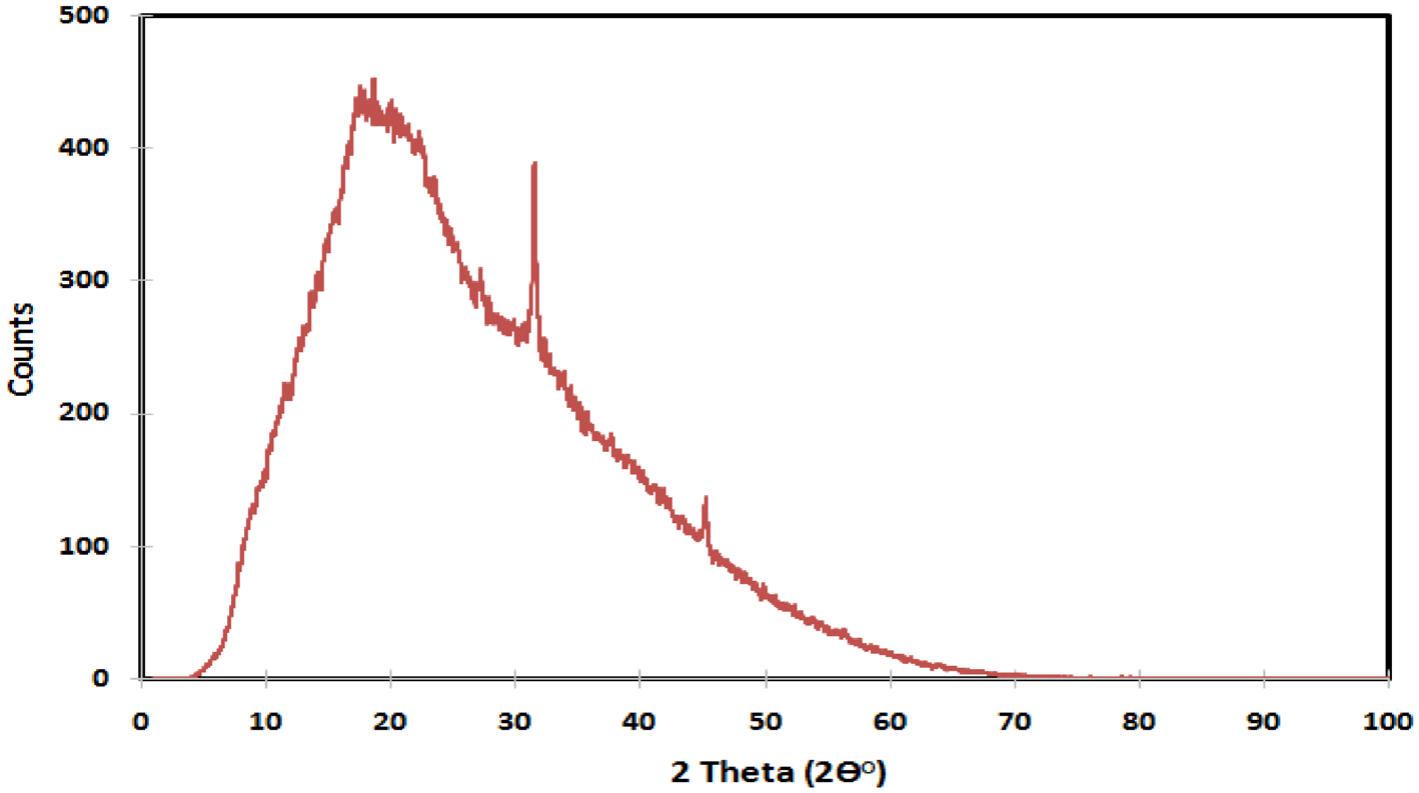
XRD of XG-g-PAA-CaCl_2_ hydrogel.

**Fig. 11:**
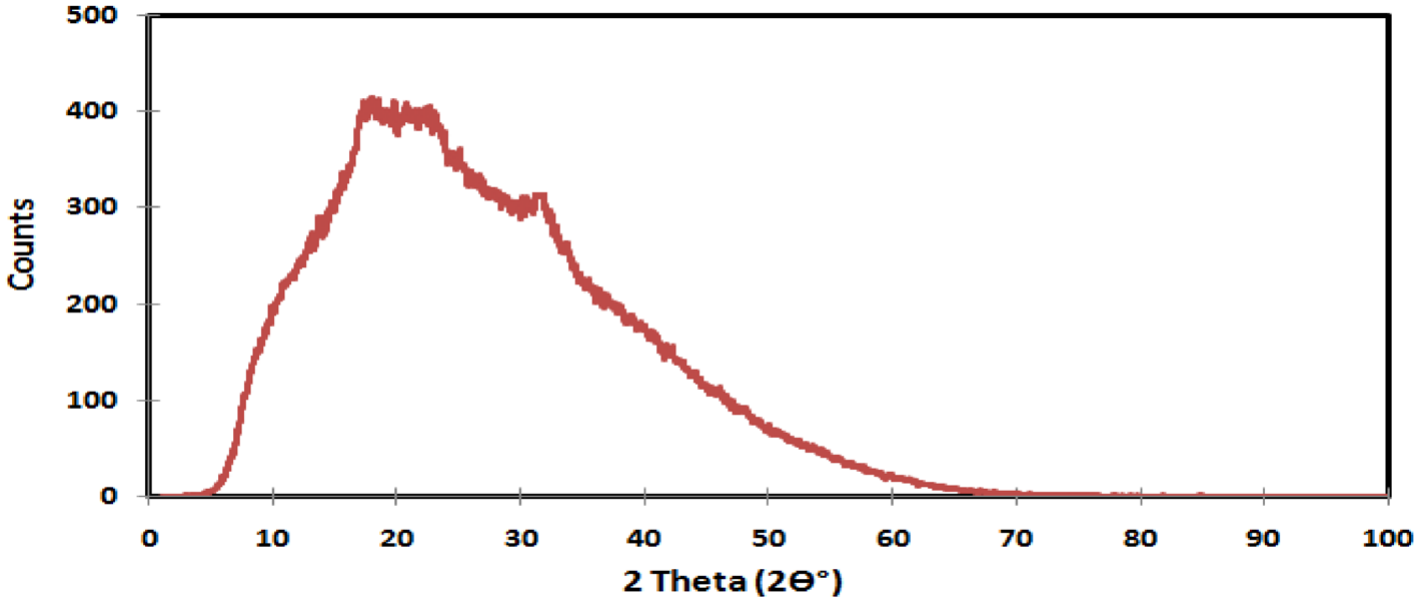
XRD of XG-g-PAA hydrogel.

XRD is considered to be a powerful tool for analyzing the properties of materials. In this study, the XRD was utilized to verify the presence and successful incorporation of CaCl_2_ within XG-g-PAA hydrogel. As can be seen that, the XRD patterns of XG-g-PAA-CaCl_2_ hydrogel (Fig. 10) displayed a characteristic sharp peak which is recognized at 2θ = 32° confirming the presence of CaCl_2_ with a crystal plane of (112), which in agreement with card number 00-049-1092 and 00-012-0056)^40^. In contrast, the XRD pattern of XG-g-PAA hydrogel (Fig. 11) showed no significant diffraction peaks of CaCl_2_.

The appearance of a characteristic diffraction peak of CaCl_2_ suggested that there is chelation between calcium ions and the chains of XG-g-PAA hydrogel revealing the successful loading of CaCl_2_^41^. To further investigate CaCl_2_ loading, sorption studies in the following section will be conducted.

### Swelling behavior of XG-g-PAA hydrogel

**Fig. 12:**
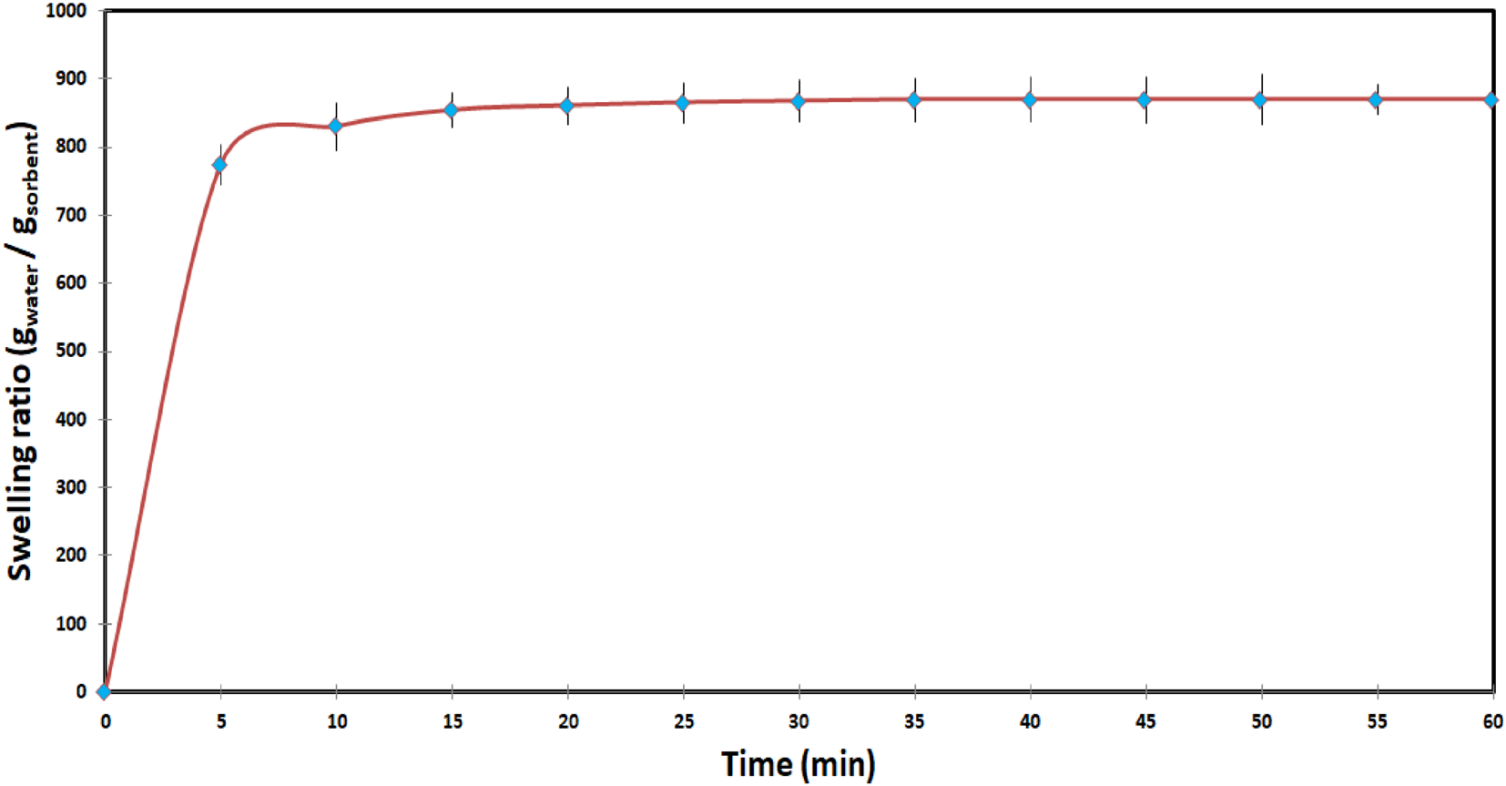
Swelling ratio (g_water_/g_sorbent_) of XG-g-PAA hydrogel in distilled water.

The water sorption of XG-g-PAA hydrogel was examined in distilled water at different time intervals and the collected data were represented in Fig. 12. It was observed that XG-g-PAA hydrogel exhibited rapid swelling, with water uptake increasing rapidly over time until reaching an equilibrium swelling capacity after a defined period. Beyond this point, there was no further increase in water absorption over time. The swelled samples reached to about 50% of their maximum swelling capacity at the first 3 min. The maximum swelling capacity was found to be 870 g_water_/g_sorbent_ for XG-g-PAA hydrogels after 35 min. The enhanced swelling capacity of the hydrogel is due to the presence of carboxylate groups, which are inherently hydrophilic and expected to expand in aqueous solutions. Additionally, the water-absorbing properties of this hydrogel result from electrostatic repulsion among the hydrophilic carboxyl groups within their network, leading to an increased osmotic pressure difference. Initially, this pressure difference facilitates rapid water absorption into the hydrogel network. Over time, more water diffuses into the hydrogel network, thereby decreasing the osmotic pressure difference between the hydrogel and its surroundings and introducing a new opposing elastic retractive force. At equilibrium, the opposing forces of osmotic pressure and elastic retraction balance each other, resulting in no further swelling^42,43^.

From the above observation, it can be concluded that the significant swelling capacity, high water retention, and insolubility of the grafted copolymer confirm the crosslinking of the samples and the formation of hydrogel.

**Fig. 13:**
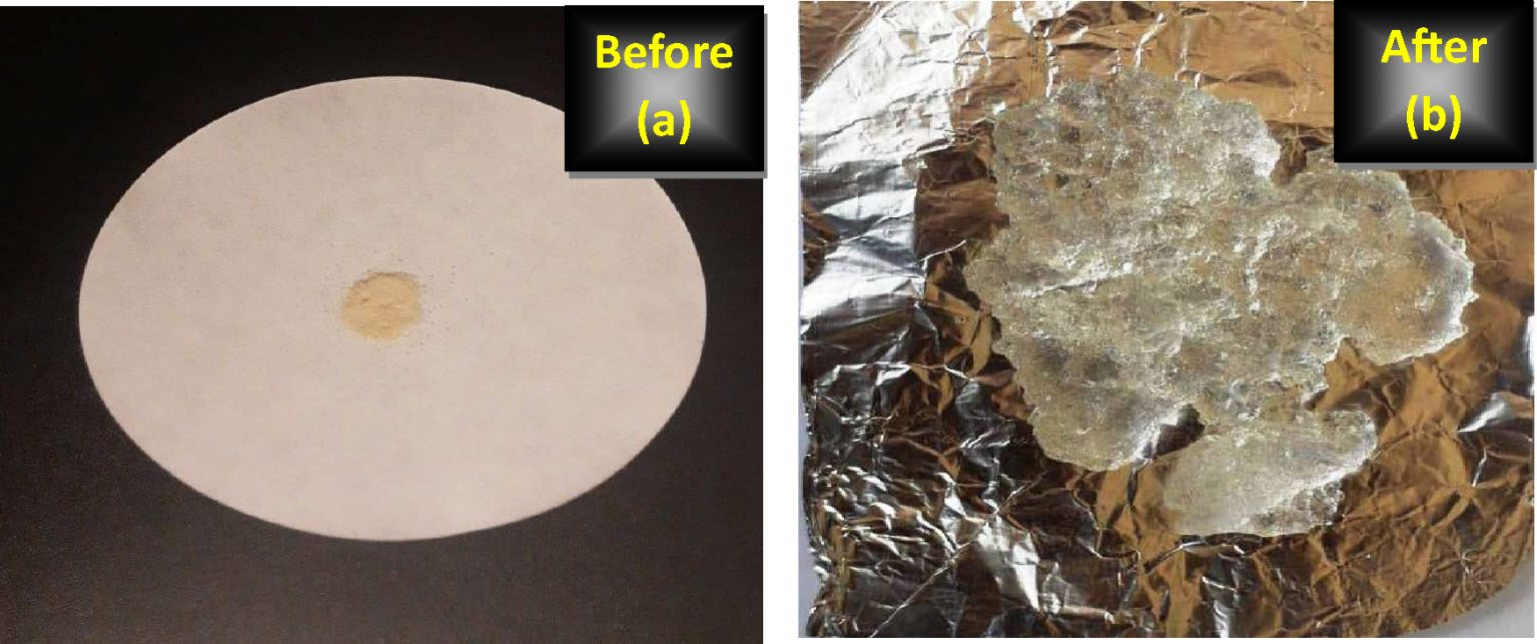
The photograph of XG-g-PAA hydrogel (a)before and (b) after the swelling test.

Figure 13 presents the photograph of XG-g-PAA hydrogel before and after the swelling test.

### Factors affecting water sorption by XG-g-PAA hydrogel

#### Crosslinking content (N-MBA)

**Fig. 14:**
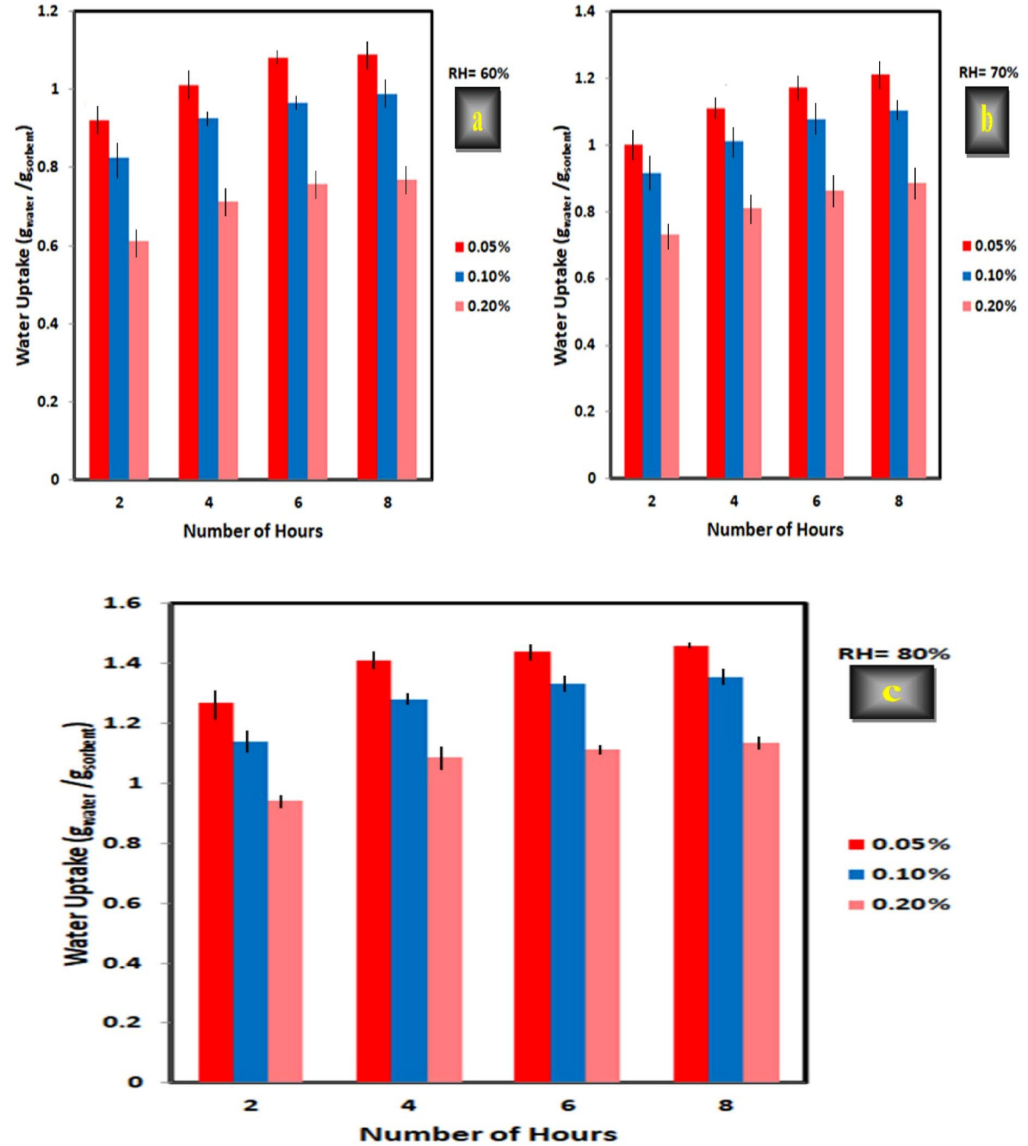
Effect of N- MBA concentration (0.05, 0.1, and 0.2%) on water sorption at various times (2, 4, 6, and 8 h) and different levels of relative humidity (a = 60, b = 70, and c = 80%) for XG-g-PAA hydrogel at constant amount of XG (1 g), AA (5 ml), and KPS (0.3 g).

From Fig. 14, it was observed that a slight increase in the concentration of the crosslinker decreased the swelling ability of the hydrogel which led to decreasing the amount of harvested water. This can be seen in XG-g-PAA (3) hydrogel which had a higher content of crosslinking agent (0.2%) compared to XG-g-PAA (1) hydrogel with a lower content of the crosslinking agent (0.05%) (Table 6). Therefore, XG-g-PAA (1) which had a low degree of crosslinking gave the higher amount of water uptake compared to XG-g-PAA (2), XG-g-PAA (3). The maximum amount of water uptake after 8 h reached to 1.46 g_water_/gs_orbent_.

The influence of increasing crosslinking can be explained as follows: higher crosslinker content leads to generation of more crosslinking points, which in turn causes an intense crosslinked structure and reduces the space between the polymer chains. This increases the rigidity of the crosslinked chains and restricts the movement and relaxation which reduces the expansion of the crosslinked hydrogel chains. As a result, increasing the quantity of N-MBA crosslinker leads to a highly rigid and inflexible crosslinked structure that cannot expand sufficiently to hold a large quantity of water. So, the swelling ability will be decreased which leads to decreasing the amount of harvested water^44,45^.

In contrast, at lower content of N-MBA (0.05% of the total weight), the degree of crosslinking is relatively low. This means that the polymeric chains are less densely interconnected; allowing them to remain more relaxed. As a result, the hydrogel structure can more easily accommodate and allow faster penetration of water into its matrix. Hence, the swelling ability will be increased and leads to an increase in the amount of harvested water vapor^46^. These results are consistent with those previously reported^47–50^. From the obtained results and the previous literatures, it can be concluded that the swelling ability has an inversely relationship to the concentration of the crosslinking agent. Thus, the synthesized hydrogel with N-MBA content of 0.05% of the total weight was selected as the optimal level for all the subsequent trials.

**Table 6:** The amount of water uptake & water collection efficiency for different series of XG-g-PAA hydrogel after 8 h at 80% RH.

Sample	XG	*N*-MBA		KPS	AA	Water uptake	Water collection Efficiency (%)
	(g)	(%)	(g)	(g)	(ml)	(gwater/gsorbent)	
XG-g-PAA (1)	1	0.05	0.0042	0.3	5	1.46	146
XG-g-PAA (2)	1	0.1	0.0084	0.3	5	1.36	136
XG-g-PAA (3)	1	0.2	0.0168	0.3	5	1.14	114

#### Monomer content (AA)

**Fig. 15:**
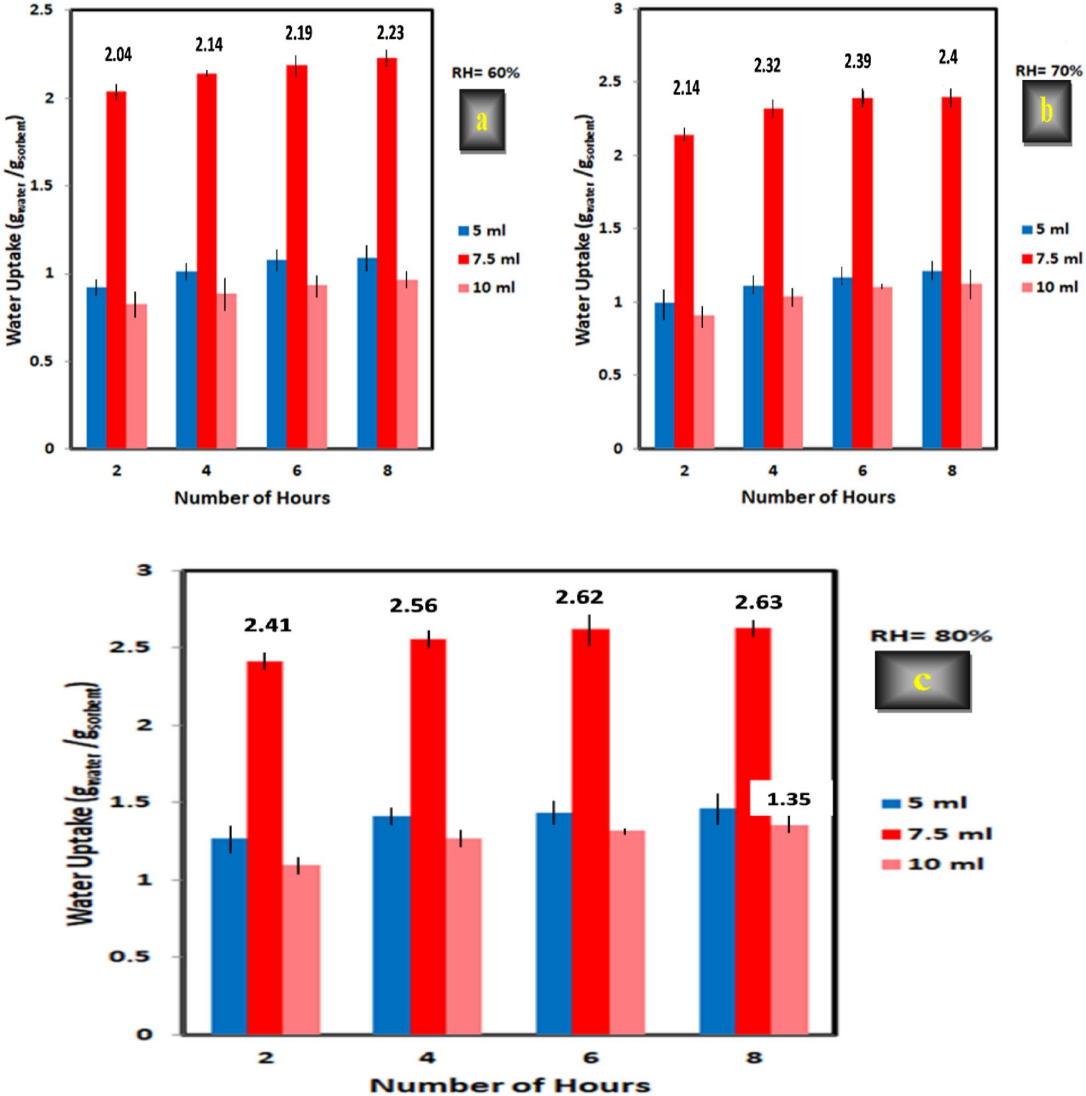
Effect of AA concentration (5, 7.5, and 10 ml) on water sorption at various times (2, 4, 6, and 8 h) and different levels of relative humidity (a = 60, b = 70, and c = 80%) for XG-g-PAA hydrogel at constant amount of XG (1 g), N-MBA (0.05%), and KPS (0.3 g).

From the obtained results, it was found that, as the amount of AA increased from 5 to 7.5 ml, the amount of water uptake in case of XG-g-PAA hydrogel increased significantly from (1.09 to 2.23) g/g, from (1.21 to 2.40) g/g, and from (1.46 to 2.63) g/g after 8 h at RH = 60, 70, and 80% respectively as shown in Fig. 15. Also. The water collection efficiency increased from 146 to 263% after 8 h at RH = 80%.

The significant increase in the amount of water uptake can be attributed to the higher amount of acrylic acid, which introduces additional hydrophilic groups such as -COO^−^ and -COOH onto the polysaccharide backbone (XG). Therefore, the presence of a greater number of ionized functional groups within the hydrogel network induces strong electrostatic repulsion between the chains which causes the expansion of the coiled chains and results in enhancing both the hydrogel swelling ability and the amount of harvested water^51,52^.

Nonetheless, after 7.5 ml, both the sorption efficiency and the amount of water harvested by the prepared hydrogel are declined significantly with increasing the amount of AA up to 10 ml and reached 1.35 g/g with water collection efficiency of 135% at RH 80% after 8 h for XG-g-PAA hydrogel as can be shown in Fig. 15.

The unexpected decreasing after reaching an optimum value could be related to the shielding effect of excess Na^+^ ions, which originates from the neutralization of carboxyl groups with NaOH which shields the carboxyl groups on the surface of the sorbent so, decreases the repulsion between them and causes a decrease in the sorption efficiency of XG-g-PAA hydrogel^53^. Previous studies have reported similar findings that are consistent with the obtained results in this study^36,47,54,55^.

From the obtained results, the synthesized hydrogel with AA amount of 7.5 ml and N-MBA concentration of 0.05% of the total weight was selected as the optimal levels for all the subsequent trials.

#### Hygroscopic salt content (CaCl_2_)

**Fig. 16:**
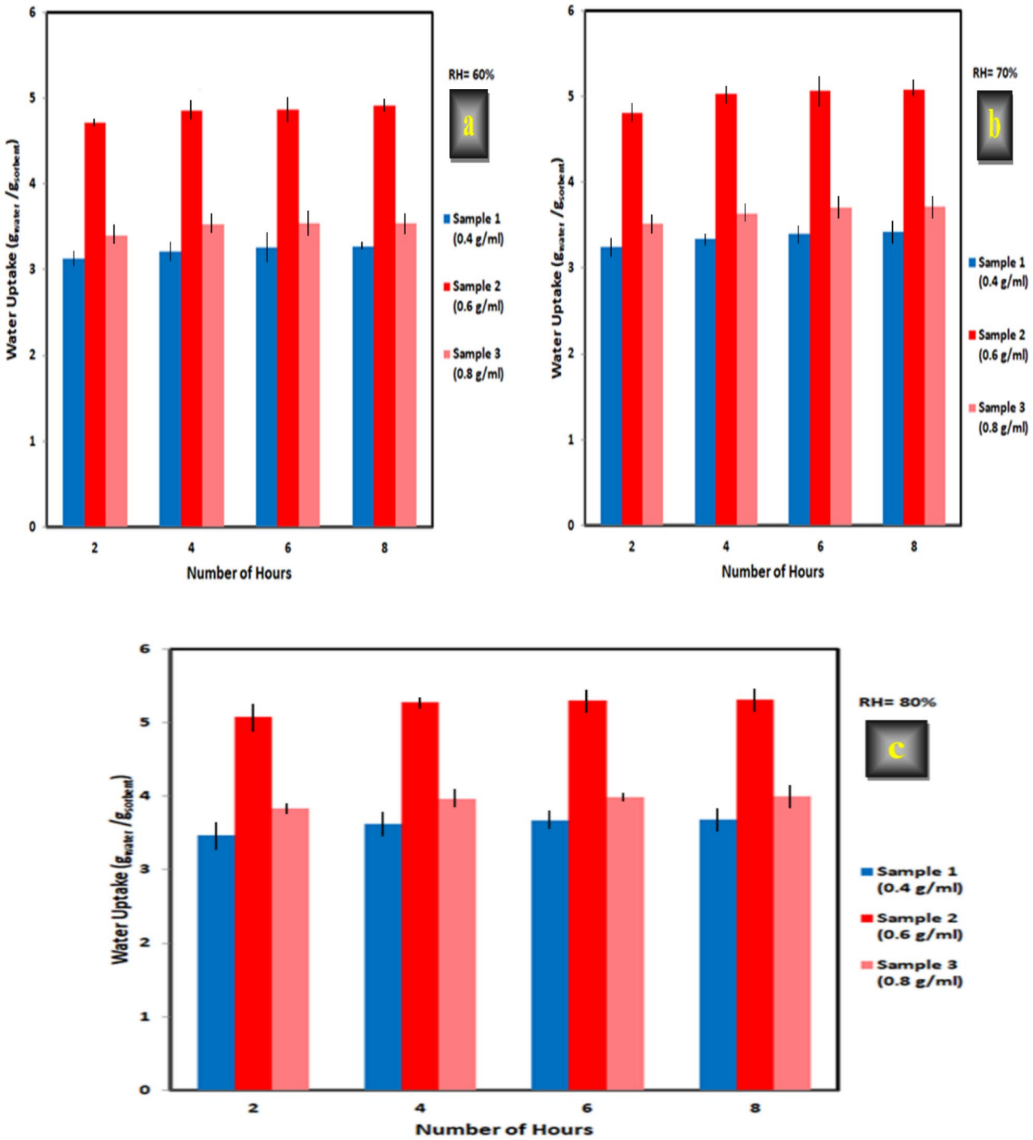
Effect of CaCl_2_ concentration (0.4, 0.6, and 0.8 g/ml) on water sorption at various times (2, 4, 6, and 8 h) and different levels of relative humidity (a = 60, b = 70, and c = 80%) for XG-g-PAA hydrogel at constant amount of XG (1 g), N-MBA (0.05%), AA (7.5 ml), and KPS (0.3 g).

As can be seen from Fig. 16 at all relative humidity levels, the sorption capacity of the synthesized hydrogel after immersion for 24 h in CaCl_2_ aqueous solution increased as the concentration of CaCl_2_ increased from 0.4 [sample (1)] to 0.6 g/ml [sample (2)]. However, it decreased for sample (3) with CaCl_2_ concentration of 0.8 g/ml. The grafted hydrogel without CaCl_2_ salt, i.e. XG-g-PAA hydrogel, exhibited the maximum sorption capacities of 2.23, 2.40, and 2.63 g_water_/g_sorbent_ for XG-g-PAA hydrogel at 60, 70, and 80% RH, respectively (Table 7). However, for sample (1), with CaCl_2_ salt concentration of 0.4 g/ml, the sorption capacity increased to 3.26, 3.42, and 3.68 g_water_/ g_sorbent_ for XG-g-PAA hydrogel at 60, 70, and 80% RH, respectively. The sorption capacity increased gradually as the concentration of CaCl_2_ increased up to 0.6 g/ml, i.e. up to sample (2), and became 4.91, 5.08, and 5.31g_water_/g_sorbent_ for XG-g-PAA hydrogel at RH = 60, 70, and 80%, respectively. The higher sorption capacity was attributed to increasing the amount of hygroscopic salt within the polymer matrix because these salts have remarkable water vapor sorption capacity^56^. Consequently, the sorption capacity increased as the concentration of CaCl_2_ salt in the polymeric network increased up to 0.6 g/ml. However, for sample (3), with CaCl_2_ concentration of 0.8 g/ml, the capacity reduced to 3.54, 3.71, and 3.99 g_water_/g_sorbent_ for XG-g-PAA hydrogel at 60, 70, and 80% RH, respectively (Table 7) which might be attributed to the coordination between carbonyl (C = O) oxygen on the molecular chain and Ca^2+^ enhances the density of physical crosslinking in the hygroscopic hydrogel, thereby reducing its performance regarding absorbing water^57^. These obtained results are in good agreement with the existing literature, where it is reported that the swelling of the polymeric matrix decreases at higher concentrations of salt^28,32,58,59^. Therefore, as long as sample (2) exhibited the highest water vapor sorption capacity, it was chosen as the optimized one and used for further detailed studies.

**Table 7:** The amount of harvested water before and after the addition of CaCl_2_ (0.4, 0.6, and 0.8 g/ml) & water collection efficiency at RH = 60, 70, and 80% for XG-g-PAA hydrogel.

Sample	XG (g)	AA (ml)	KPS (g)	*N*-MBA (%)	CaCl_2_ (g/ml)	RH = 60%		RH = 70%		RH = 80%	
						Water uptake (g/g)	Water collection efficiency (%)	Water uptake (g/g)	Water collection efficiency (%)	Water uptake (g/g)	Water collection efficiency (%)
XG-g-PAA hydrogel (0)	1	7.5	0.3	0.05	0.0	2.23	223	2.4	240	2.63	263
XG-g-PAA hydrogel (1)	1	7.5	0.3	0.05	0.4	3.26	326	3.42	342	3.68	368
XG-g-PAA hydrogel (2)	1	7.5	0.3	0.05	0.6	4.91	491	5.08	508	5.31	531
XG-g-PAA hydrogel (3)	1	7.5	0.3	0.05	0.8	3.54	354	3.71	371	3.99	399

#### Water release experiment

**Fig. 17:**
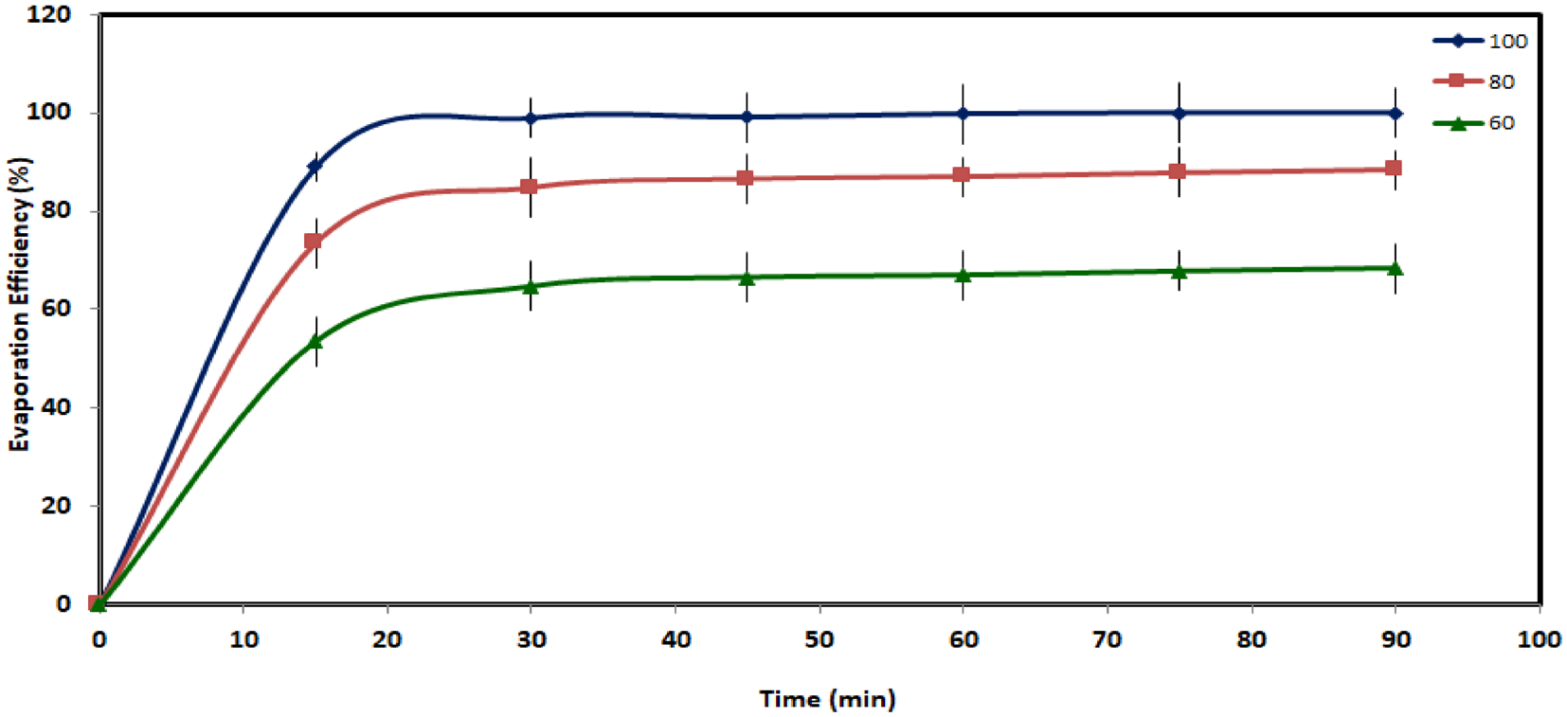
The Evaporation Efficiency of the hydrated XG-g-PAA-CaCl_2_ hydrogel at various time intervals and different temperatures (60. 80. and 100 °C).

From the obtained Fig. 17 for XG-g-PAA-CaCl_2_ hydrogel, it can be observed that the hydrogel showed a rapid and remarkable water release (which represented in terms of % of evaporation efficiency) within the first 30 min. Subsequently, the rate of release slowed as the water content decreased. After calculations, it was recorded that the percentage of evaporation efficiency in the first 30 min of release were 64.6, 84.7, and 98.5% at 60, 80, and 100 °C respectively for XG-g-PAA-CaCl_2_ hydrogel.

As temperature increased, XG-g-PAA-CaCl_2_ hydrogel released water more rapidly, achieving 100% release of sorbed water within 90 at 100 °C. This highlights the potential for utilizing natural sunlight to facilitate water release, enhancing water harvesting capabilities in diverse environmental conditions.

#### Reusability test

Reusability is considered to be a crucial factor in assessing the overall performance of materials. For a sorbent to be suitable for various industrial applications, it must demonstrate the capability to undergo multiple cycles of sorption and regeneration without degradation. Consequently, the water harvesting cyclic durability and stability of the synthesized CaCl_2_ incorporated hydrogels, i.e. XG-g-PAA-CaCl_2_ hydrogel, were assessed by subjecting them to 12 consecutive cycles of water vapor sorption and regeneration.

**Fig. 18:**
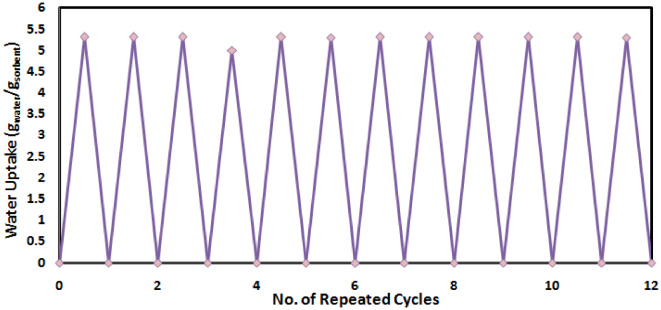
The reusability test for the sorption- desorption performance for XG-g-PAA-CaCl_2_ hydrogel during 12 cycles.

It is evident from Fig. 18 that the water vapor uptake and release capabilities of XG-g-PAA-CaCl_2_ hydrogel show no significant structural deformation after multiple sorption-desorption processes, indicating that XG-g-PAA-CaCl_2_ hydrogel had good structural performance stability and possess long-term operational stability.

The high sorption capacity observed during each sorption/regeneration cycle for XG-g-PAA-CaCl_2_ hydrogel also indicates that the deliquescent salt, CaCl_2_, remained embedded within the polymer matrix without leaching out which is one of the significant achievements of this thesis work.

According to the above experiments, it can be concluded the fact that, XG-g-PAA-CaCl_2_ hydrogel can be effectively used repeatedly as a sorbent for AWH application.

## Conclusions

From the present research work, we can conclude the followings:

1. A novel supersorbent hydrogels (XG-g-PAA) based on natural polysaccharide polymer were successfully synthesized through grafting of partially neutralized AA onto the backbone of XG using of N, N′-methylene-bis-acrylamide (N-MBA) as a crosslinker and potassium persulfate (KPS) as an initiator using microwave assisted method.
2. Using the microwave method, % G and % GE were rapidly increased as the amount of AA increased and reached to the maximum value than the conventional method, indicating the significant role of microwave radiation in facilitating the grafting of AA onto XG.
3. The swelled samples showed a higher value of water uptake and reached to about 50% of their maximum swelling capacity at the first 3 min and exhibiting maximum swelling capacity of 870 g_water_ /g_sorbent_ for XG-g-PAA after 35 min.
4. Furthermore, the effect of crosslinker concentration, monomer content, and hygroscopic salt concentration on the amount of harvested water by the synthesized hydrogel were investigated and it was found that:
  - The amount of water vapor harvested by XG-g-PAA hydrogel is increased as the concentration of crosslinker (N-MBA) decreased from 0.2 to 0.05% of the total weight and reached to 1.46 g_water_/g_sorbent_ at RH = 80%.
  - The amount of water vapor harvested by XG-g-PAA hydrogel is increased as the amount of monomer (AA) increased from 5 to 7.5 ml and reached to 2.63 g_water_/g_sorbent_ at RH = 80%. Further increase in the amount of AA (i.e. 10 ml) resulted in decreasing the amount of harvested water to reach 1.35 g_water_/g_sorbent_ at RH = 80%.
  - The addition of hygroscopic salt (CaCl_2_) enhanced the swelling ratio of the synthesized hydrogel. Also, as the concentration of CaCl_2_ increased from 0.4 to 0.6 g/ml, the amount of atmospheric water captured by XG-g-PAA hydrogel increased rapidly and reached to 5.31 g_water_/g_sorbent_ at RH = 80%. However, at a higher concentration of CaCl_2_ (0.8 g/ml), the water uptake decreased and became 3.99 g_water_/g_sorbent_ at RH = 80%.

5. Finally, desorption and reusability studies were performed and it was observed that the water harvested from the hydrogel could be released easily, with over 94% of the sorbed water being rapidly released. While, the reusability study showed that XG-g-PAA hydrogel had excellent reusability without noticeable capacity fading.
6. The use of deliquescent salt within the hydrogel network enhances its ability to interact effectively with water molecules, leading to efficient water harvesting without the need for extra energy. This technology offers a practical and economical solution to address global environmental challenges, providing a sustainable source of clean water for households, industries, and agriculture by extracting water from the air.

## Supplementary Information

Below is the link to the electronic supplementary material.

Supplementary Material 1

## Data Availability

The data will be available from the corresponding author upon request.
