# Supplementary material for: Recyclable and eco-friendly xanthan gum-based hygroscopic hydrogel for atmospheric water harvesting
Source: Sci Rep. 2025 Nov 17;15:40203. doi: 10.1038/s41598-025-23971-3 (PMC12623978; doi:10.1038/s41598-025-23971-3)
Supplement: Supplementary file 1 — Supplementary Material 1 [file 41598_2025_23971_MOESM1_ESM.docx]

**Recyclable And Eco-Friendly Xanthan Gum-Based Hygroscopic Hydrogel for Atmospheric Water Harvesting**

**A’laa Mohamed Safwat^*1^, Mohamed Abd-Elzaher^1^, I. H. Saleh^2^, Moataz Soliman^2^, Wagih Abdel‑Alim Sadik^2^.**

**1.** Department of Basic and Applied Sciences, Faculty of Engineering, Arab Academy for Science, Technology, and Maritime Transport, Egypt.

**2.** Institute of Graduate Studies & Research, Alexandria University, Egypt.

Figure S1: The photographs of the uncropped XG-g-PAA hydrogel.
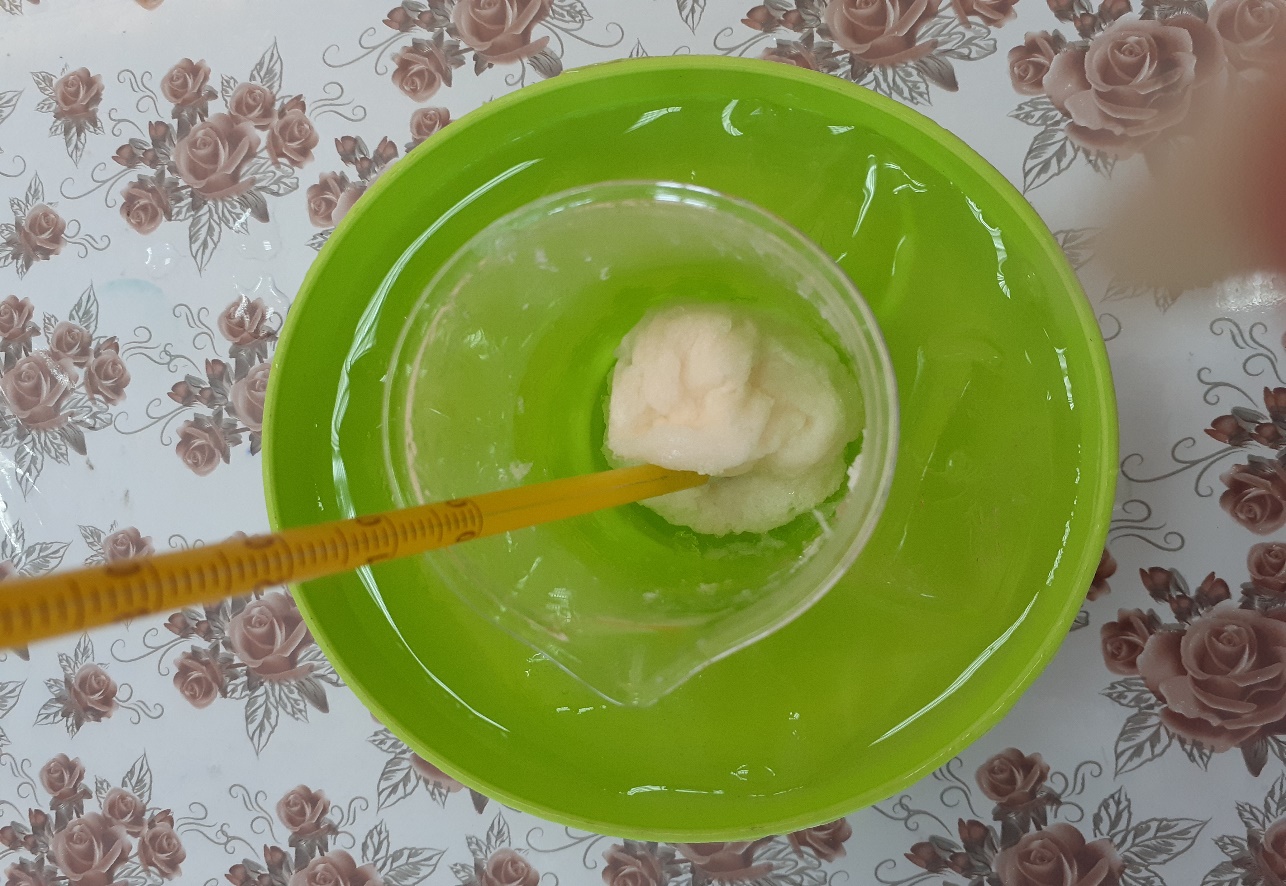


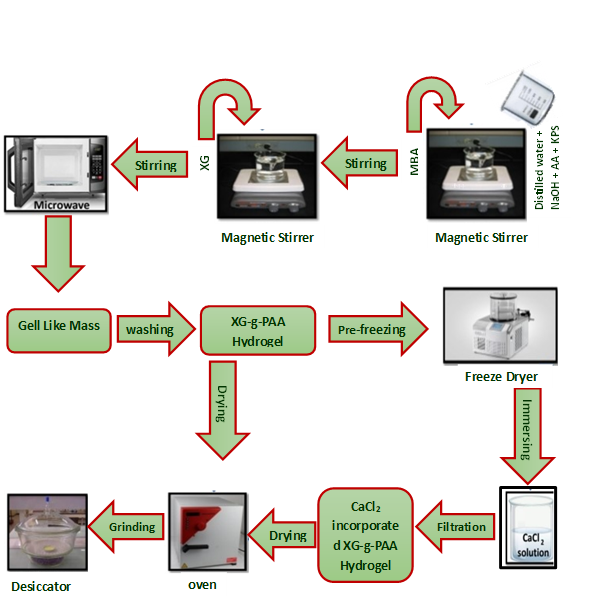


**Figure S2:** The preparation procedures for XG-g-PAA and CaCl_2_ incorporated XG-g-PAA hydrogel.
